# Most industrialised countries have peaked carbon dioxide emissions during economic crises through strengthened structural change

**DOI:** 10.1038/s43247-023-00687-8

**Published:** 2023-02-21

**Authors:** Germán Bersalli, Tim Tröndle, Johan Lilliestam

**Affiliations:** 1 Energy Transitions & Public Policy group, Research Institute for Sustainability—Helmholtz Centre Potsdam, Potsdam, Germany; 2 Climate Policy Lab, Institute for Environmental Decisions, ETH Zürich, Zürich, Switzerland; 3 Faculty of Economics and Social Sciences, University of Potsdam, Potsdam, Germany

**Keywords:** Climate-change mitigation, Environmental economics, Economics, Environmental studies

## Abstract

As the climate targets tighten and countries are impacted by several crises, understanding how and under which conditions carbon dioxide emissions peak and start declining is gaining importance. We assess the timing of emissions peaks in all major emitters (1965–2019) and the extent to which past economic crises have impacted structural drivers of emissions contributing to emission peaks. We show that in 26 of 28 countries that have peaked emissions, the peak occurred just before or during a recession through the combined effect of lower economic growth (1.5 median percentage points per year) and decreasing energy and/or carbon intensity (0.7) during and after the crisis. In peak-and-decline countries, crises have typically magnified pre-existing improvements in structural change. In non-peaking countries, economic growth was less affected, and structural change effects were weaker or increased emissions. Crises do not automatically trigger peaks but may strengthen ongoing decarbonisation trends through several mechanisms.

## Abstract (web summary)

In most developed economies where decarbonisation processes are already in place, economic crises have coincided with a peak and subsequently declining emissions as a result of lower carbon and energy intensities, according to a statistical analysis of 45 countries between 1965 and 2019.

## Introduction

Economic disruption associated with the Covid-19 pandemic and the current energy and economic crisis related to the war in Ukraine emphasises the importance of understanding and managing crises’ immediate and lasting effects on decarbonisation^1–5^. To reach the Paris Agreement’s targets, every country must first peak, then reduce and eventually eliminate its carbon dioxide (CO_2_) emissions by mid-century^6^. There is rich literature on emissions drivers^7–9^ and decoupling, showing that many countries—particularly in Europe—have peaked their CO_2_ emissions and started to reduce them. However, the reasons why peaks happen at a particular time remain unclear, including the role of economic crises: empirical evaluations are scarce and suggest that crises have had no lasting effects on decarbonisation^1,10^. Yet, conceptual analyses from different disciplines indicate that crises may trigger long-lasting changes in national economies and energy systems through structural change: shifts in the economic structure and the technologies used, particularly energy technologies, due to economic forces or public policy (Supplementary Note 1.1). In this article, we examine to which extent past crises have impacted structural change contributing to national (territorial-based) emissions peaks.

Theoretically, there are several reasons to expect a deep economic crisis to support the structural change needed for decarbonisation through interactions between political, economic, and social factors (Supplementary Note 1.2). Schumpeterian economists have pointed to “creative destruction”^11^ as an essential driver of structural change. During economic crises, the least efficient assets (or entire industries) may collapse and not come back again during recovery because they are replaced by new, more efficient assets or activities. Since these “creatively destroyed” assets are often also the least energy- or carbon-efficient ones, the result is structural change and a lasting reduction of emissions. Politically, crises may open windows of opportunity for action and trigger critical junctures in energy and climate policy^12^, allowing for paradigmatic policy shifts that are hard or impossible during normal times. Crises may also allow the implementation of green recovery packages following a Green Keynesianism approach^13–16^. From a transition studies perspective, external landscape shocks—like economic crises—can destabilize existing socio-technical regimes, thereby enabling regime change and a transition to a new, lower-carbon system^17^. Hence, there are substantial theoretical arguments suggesting that economic crises may, because they are disruptive, be conducive to the type of structural, long-term change needed for decarbonisation.

Nevertheless, other scholars argue that crises can have negative effects by increasing uncertainty and thus hampering private investments, especially in new and thus risky technologies^18,19^. This could be particularly fatal for investment-intensive decarbonisation processes. Further, crises can shift political priorities from solving long-term issues like climate change to the immediate socio-economic impacts of the crisis^20^, and restoring the economy as it was before the recession^21^. Such effects would thus mainly work to preserve the economy and be detrimental to structural change—a crisis may briefly reduce emissions due to lower economic activity but not trigger lasting effects. Thus, the expected effects of crises are contested and could be supportive or detrimental to decarbonisation.

Empirically, evaluations of the effects of crises on emissions are scarce and show inconclusive results (Table S1, Supplementary Note 1.3). The first assessments of the Covid-19 pandemic impacts suggested that the reduction in CO_2_ emissions was substantial in 2020^10,22^ but is rapidly rebounding as countries return to previous growth trends^1,23^; this V-shaped emission trajectory was observed for the Global Financial Crisis (GFC), with only short-lived emission reductions of little importance for decarbonisation^24^. However, later case studies for southern European countries found lasting effects of the GFC. Results of two studies comprising larger samples of countries also suggest that past crises have had a substantial impact on CO_2_ and methane emissions^25^, or on decoupling between CO_2_ emissions and economic growth^26^. Importantly, no one of these studies systematically investigated the effects of crises on structural change, which is paramount for decarbonisation in the long run.

The broader literature examining CO_2_ emissions drivers^7^ showed that increases in overall activity levels invariably led to increases in emissions, while improvement in energy intensity was a key reducing driver (Supplementary Note 1.4). A review of CO_2_ drivers between 1990–2018^8^ revealed moderate decarbonisation of energy systems in Europe and North America, driven by fuel switching and the increasing penetration of renewables. By contrast, fossil-based energy systems have continuously expanded in rapidly industrialising regions, only very recently slowing down their growth. Papers focusing on decoupling have shown that many countries—particularly in Europe—have peaked their CO_2_ emissions and achieved absolute decoupling (GDP growths while emissions decrease), both for production- and consumption-based emissions^27–30^. Still, these studies did not evaluate the impacts of economic crises and have called for more research on structural drivers and emissions peaks^27^.

Here, we link two essential streams of the empirical literature on decarbonisation: assessment of (i) CO_2_ emissions drivers and (ii) the impacts of crises on structural change toward decarbonisation. We assess the extent to which past crises have impacted carbon and energy intensities (structural change effect) and the economic (GDP effect) drivers of CO_2_ emissions leading to emission peaks. We investigate this in the 45 countries that are part of the OECD and the G20 (or both) in 1965–2019: Are national CO_2_ emission peaks associated with economic crises? And if so, which economic and structural mechanisms explain such crisis-related emissions peaks? Through Kaya decomposition and statistical analysis, we examine countries that have peaked and such that have not, showing that in 26 of 28 countries that have peaked, the peak occurred just before or during an economic crisis through the combined effect of lower post-crisis GDP growth and intensified structural change leading to decreased energy or carbon intensity, or both. Changes in the GDP effect post-crises are statistically significant for both peak and non-peak countries groups, but it was stronger in the former. In contrast, the structural change effect is significant only for the peak group. We conclude that crises do not automatically trigger emissions peaks and structural change, but they have contributed to emissions peaks in developed economies, especially where national decarbonisation processes had already begun when the crisis struck.

## Results

### Global CO_2_ emissions and the temporality of national emissions peaks

**Fig. 1:**
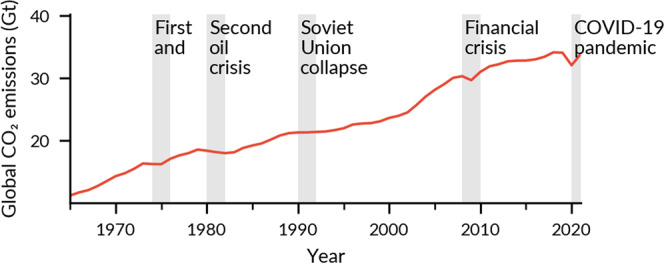
CO_2_ emissions and major economic crises. Global CO_2_ emissions from fossil fuel combustion. The grey shades represent the five biggest economic crises in 1965–2021. Source:^31^.

**Fig. 2:**
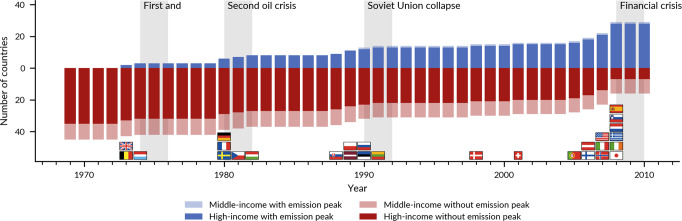
Emissions peaks and economic crises. Number of countries that have and have not peaked CO_2_ emissions (the year with the highest rolling average of the five past years). OECD and G20 countries. The flag indicates the peak year of the respective country. Flags designed by OpenMoji. Source:^31^.

Our results show that past crises decreased global emissions only weakly and during short periods (Fig. 1), but also that crises have had lasting effects on specific regions and countries, contributing to national CO_2_ emissions peaks (Fig. 2).

In the last 50 years, global CO_2_ emissions have steadily increased, punctuated by small dips in the curve (e.g., −2.1% in 2009^31^) during severe economic crises. These events, such as the two oil crises (1973–75 and 1979–81), the collapse of the Soviet Union (1989–1991), and the GFC (2007–09), affected the global emissions curve, but only as short-lived dents, suggesting that economic crises do not have an important effect on global CO_2_ emissions (Fig. 1). The global effect is reduced as countries are unevenly affected: some suffer from deep recessions while others continue growing. Even the GFC, the then largest global economic crisis since the 1930s, triggered a recession only in 100 countries in 2008–09^32^, and most of them returned to a growth path quickly. The Covid-19 crisis, which caused a recession in 142 countries and reduced the global GDP by 3.2% in 2020^32^, reduced CO_2_ emissions by −5.9% in 2020, followed by a rebound of 5.6% in 2021^31^.

However, those short-lived global dips hide significant developments in national economies. Of the 45 OECD and G20 countries we investigate, 28 have passed their national energy-related CO_2_ emissions peaks, with the first peaks happening in the 1970s (Supplementary Note 2). Because all but two countries have also experienced GDP growth since their peak emissions year (to 2019), this shows that absolute decoupling is possible, even over long periods of up to 50 years (Supplementary Note 3). This is important but neither new nor surprising^8,29^. Yet, our results also show that emissions peaks generally have occurred during an economic crisis (Fig. 2), especially the four large global crises (the two oil crises, the Soviet Union Collapse, and the GFC). Of the 28 countries that have peaked, 26 did so just before (0–2 years) or during a crisis. Only Denmark (1998) and Switzerland (2001) peaked emissions seemingly unrelated to a deep crisis (Supplementary Note 4).

During the first oil crisis in 1973/75, which triggered an economic crisis especially in Western economies, the first countries peaked, all of them among the hardest-hit western European countries (the UK, Belgium, and Luxembourg). Five further countries (Germany, France, Sweden, Hungary, and the Czech Republic) peaked during the second oil crisis and kept decreasing emissions post-crisis. The dissolution of the Soviet Union (1989–1991) coincided with emission peaks in most ex-Soviet and several eastern European countries. Finally, a range of industrialised countries, especially in hard-hit Europe but also the US and Japan, peaked emissions just before the onset of the GFC (Fig. 2). In peak countries, emissions sometimes increased in specific years after the peak but never reached pre-peak emissions levels again. Fig. S2 (Supplementary Note 6) shows the decomposed time series for all countries and the whole period.

There is, thus, a temporal connection between economic crises and national peak emissions. The decomposition analysis presented in the following sections aims to explain how crises impacted CO_2_ emissions drivers in peak and non-peak countries.

### The impact of crises in peak-and-decline countries

**Fig. 3:**
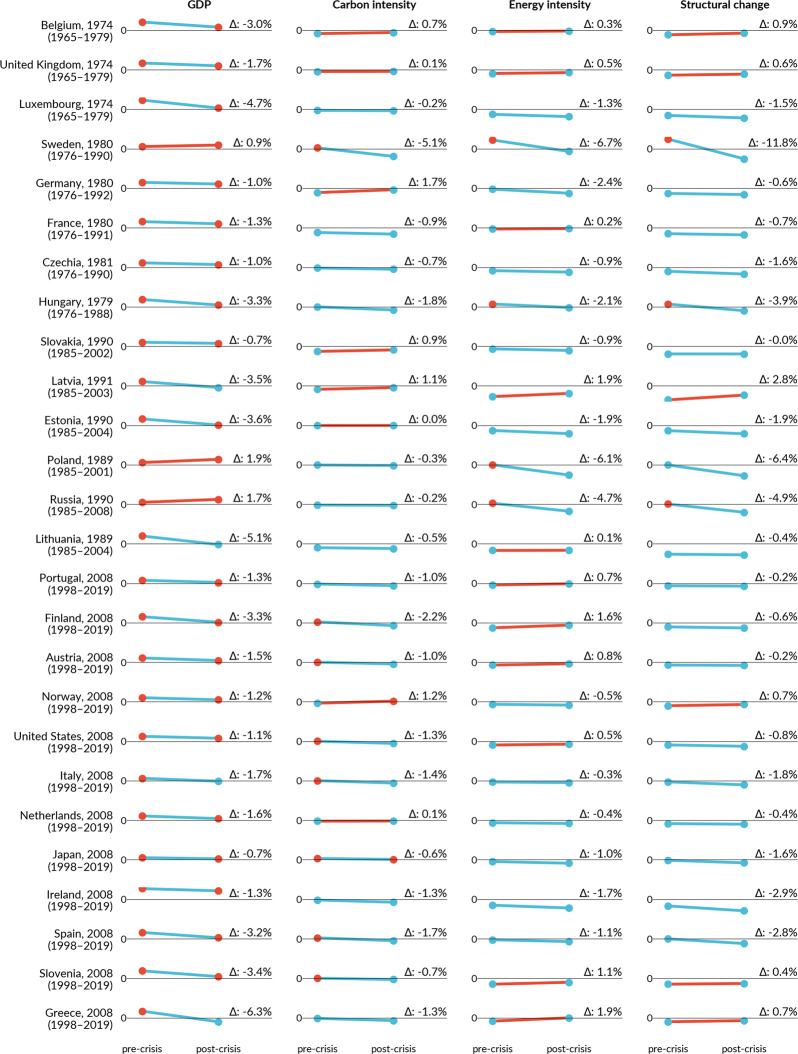
Decomposition of CO_2_ emission drivers in peak-and-decline countries. Changes in growth rates (% per year) of contribution factors to carbon emissions before (first point) and during and after (last point) the economic crisis associated to the CO_2_ peak, based on the Kaya identity and multiplicative decomposition. GDP includes population and GDP per capita factors. Structural change is the change of combined energy and carbon intensity factors. Red (blue) circles mean that the factor increased (reduced) emissions; red (blue) lines indicate that the change from pre- to post-crisis was negative (positive) for decarbonisation. The year after the country name denotes the first year of recession and the period below the country name denotes the entire analysed period. Source:^31,32,57^.

The decomposition analysis and statistical tests show two effects explaining the peak timing. First, GDP growth decreased by 1.5 (–3.3 to −1.1) percentage points (*p* < 0.01) in the post-crisis period (Table 1). In 23 of 26 countries with a crisis-related peak, the contribution of the GDP effect to CO_2_ emissions was lower post-crisis than before (Fig. 3, GDP blue lines). However, all countries eventually returned to economic growth, meaning that GDP continues to increase emissions, but slower than before. Only in Lithuania, Latvia (Soviet crisis), Greece and Italy (GFC), which experienced deep and long recessions after the respective peak, did the GDP effect work to slightly decrease emissions in the post-crisis period (Fig. 3, GDP blue circles).

**Table 1:** Change of GDP effect and structural change (SC) effect before and after crises.

	*N*	Median	q25	q75	Test Statistic_V	*p*
GDP no-peak	99	−0.010	−0.024	0.001	1073	0.000
GDP peak	26	−0.015	−0.033	−0.011	34	0.000
GDP all	152	−0.012	−0.027	0.000	2001	0.000
SC no-peak	97	−0.002	−0.022	0.013	2023	0.102
SC peak	26	−0.007	−0.018	−0.001	70	0.003
SC all	150	−0.002	−0.019	0.013	5053	0.126

Wilcoxon signed-rank test results for peak, non-peak, and all (45) countries in the four global crises (two oil crises, Soviet Union Collapse, Global Financial Crisis). Alternative hypothesis: contribution factor decreased in the post-crisis period. Source:^31,32,57^.

Second, the 26 countries have seen a structural change towards a sustained lower level of emissions (negative growth of combined energy and carbon intensities), and after the peak-related crisis, such effect intensified by −0.7 (−1.8 to −0.1) percentage points (*p* < 0.01). In most cases (17 of 26), pre-existing structural change effect trends intensified during and after crises, leading to faster reduction in energy intensity, carbon intensity or both. In three cases, these trends bent, meaning that they were increasing emissions before the crises and decreasing them after. In six countries, the path of technological change was stronger pre- than post-crises, but still, the structural change effect worked to keep reducing emissions (Fig. 3, Structural change, blue circles). Hence, in countries that have peaked, structural changes intensified by or coinciding with an economic crisis and lower GDP growth explain the peaks and lasting state of absolute decoupling (Supplementary Note 3).

### Mechanisms explaining structural change during crises

Our results show that crises have intensified structural change in peaking economies, most often by magnifying already ongoing improvement trends in energy and/or carbon intensity, and sometimes by shifting trends from increasing to decreasing intensities. These changes result from three interrelated mechanisms.

**Fig. 4:**
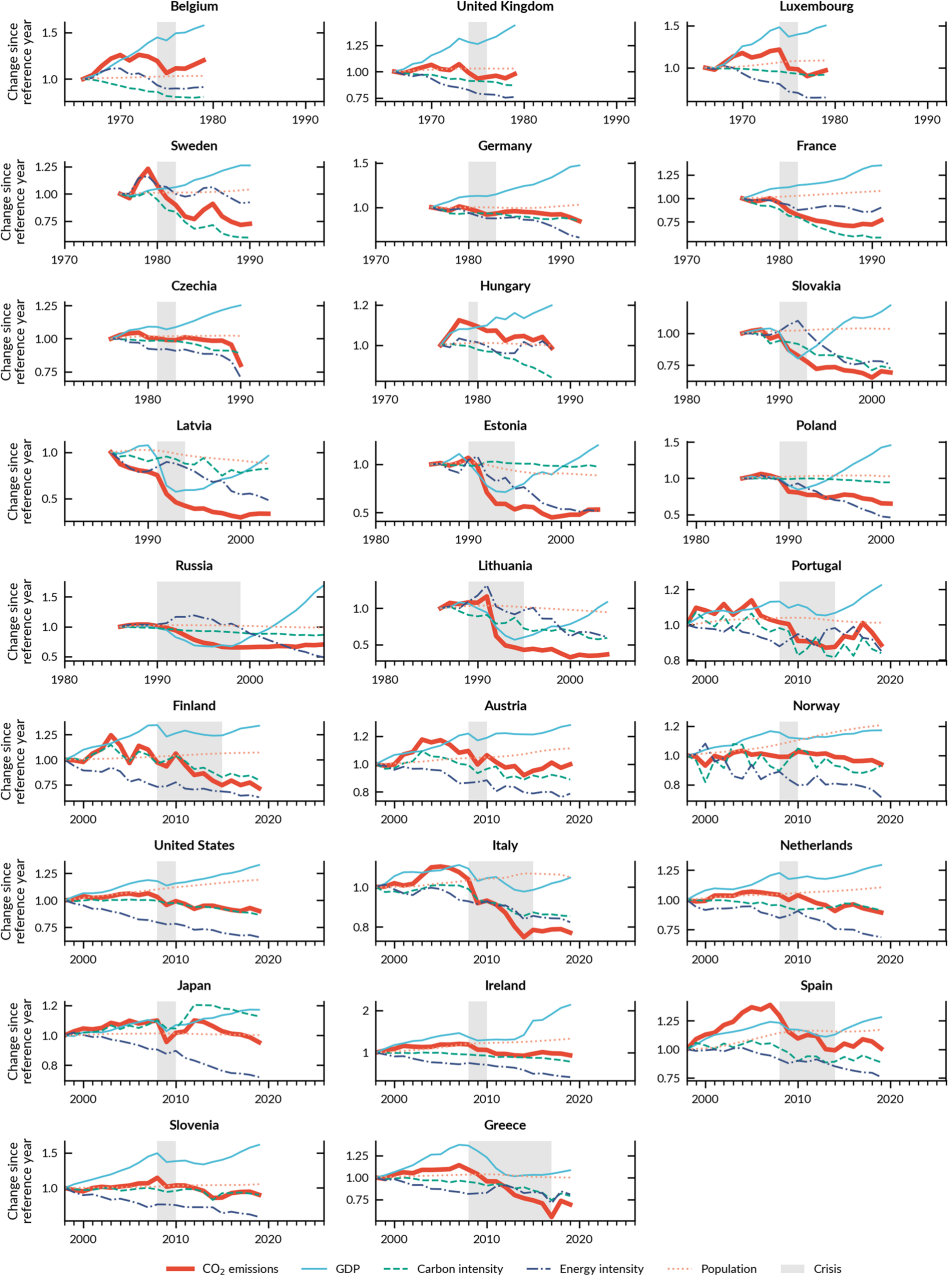
Crises & CO_2_ emission drivers. CO_2_ emissions and cumulative decomposed emission drivers from the Kaya identity; 26 countries that peaked emissions around economic crises. Charts for all countries and years in Supplementary Note 6. Sources:^31,32,57^.

The first mechanism consists of energy efficiency measures taken by governments and firms to respond to higher energy prices or deteriorating economic conditions. This is particularly strong during the oil crises. The countries that peaked in that period experienced substantial improvement in energy intensity (first two rows in Fig. 4). Responding to supply constraints and price hikes, governments implemented measures to reduce the consumption of expensive imported fuels and address industrial efficiency specially across export-oriented sectors^33,34^. Beyond public policies, firms also respond to crises and trigger new market trends, such as the shift towards smaller and more efficient cars during the oil crises, especially in the Western European and Japanese automotive industries^35,36^. Similar positive effects on energy efficiency occurred in several Western countries during the GFC (bottom four rows in Fig. 4) (e.g. Italy^37^, Ireland^37^, Japan^38^), supported by pre-existing policies^28^ and (modest) green recovery funds dedicated to energy efficiency measures^39^. In Japan, the post-GFC period overlaps with the Fukushima disaster in 2011, which also strongly impacted its energy system.

The second mechanism, also affecting energy intensity, comprises changes in economic structure due to the decline of energy/carbon-intensive industries and the rise of less energy-intensive ones post-crisis, driven by economic and sometimes political forces. When the economy recovers, it does not recover to its previous state but sees a shift to less energy- or carbon-intensive assets (e.g. modern, efficient production lines) or activities (e.g. service sector instead of manufacturing). In ex-communist countries, energy intensity decreased substantially during the 1990s and early 2000s (Fig. 4) due to economic transformation^40^; for example, Russia experienced a long recession in the 1990s followed by a booming recovery in the 2000s, during which the GDP share of industry fell from 45 to 30% (1990–2008)^32^. In Poland, structural change during the early 1990s included privatisation or shut-down of the most inefficient enterprises and cutting high subsidies to energy consumption^41^; GDP boomed since 1992 while emissions did not follow (Fig. 4). In Spain, among the hardest-hit countries during the GFC and the following Euro crisis, the effects on industry were strong, with the sectoral share of GDP falling from 26% in 2007 to 20% in 2015; particularly the construction industry collapsed and never recovered to pre-crisis levels^42^. The Spanish return to growth thus happened in other, less carbon- and energy-intensive sectors.

The third mechanism consists of changes in the energy mix, leading to reduced carbon intensity, triggered by new market conditions or policy changes. The first oil crisis had a long-lasting effect on the energy mix particularly in Western Europe, for example through large-scale deployment of nuclear power in several countries; this crisis also triggered interest in nascent renewable energy technologies, leading to the first larger-scale R&D programmes, although initially little deployment^33^. The second oil crisis provided new reasons to confirm those developments. For example, the French Messmer nuclear programme implemented in response to the first oil crisis put France on a path to largely CO_2_-free electricity from the late 1970s^43^, and the resulting fuel switch from oil to nuclear led to the French emission peak in 1980 (Supplementary Note 5). Similar energy policy developments explain the Swedish emissions peak in 1979, resulting in a shift from oil to bioenergy and nuclear power^44^. Furthermore, during and following the GFC, most (10 of 12) peaking countries improved their carbon intensity trends, especially as coal power was pushed out of power systems due to changing market conditions and dedicated policy. The GFC recovery packages did not have a major effect on the energy mix, as they were focused on end-use efficiency and the car sector^39^. Further, the deployment of renewable energy continued through and after the crisis, and accelerated in some countries, such as Italy and the US^45^. Despite the deterioration of the fiscal situation during the GFC, most governments continued to support renewable energy, which also benefitted from lower interest rates resulting from expansive monetary policy following the crisis^46,47^.

**Fig. 5:**
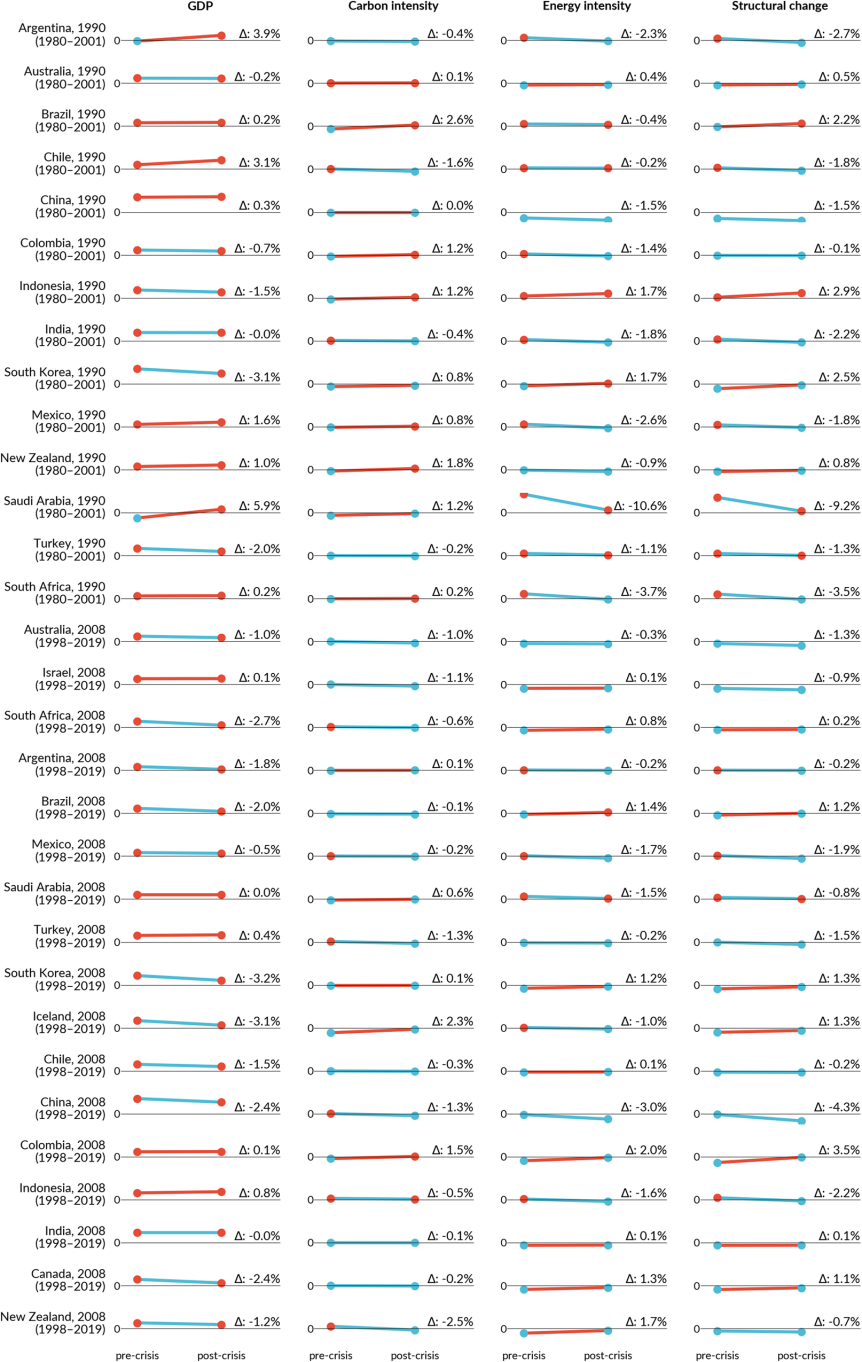
Decomposition of CO_2_ emission drivers in non-peaking countries following the GFC and Soviet crisis. Changes in growth rates (% per year) of contribution factors to carbon emissions before (first point) and during and after (last point) the economic crisis associated to the CO_2_ peak, based on the Kaya identity and multiplicative decomposition. GDP includes population and GDP per capita factors. Structural change is the change of combined energy and carbon intensity factors. Red (blue) circles mean that the factor increased (reduced) emissions; red (blue) lines indicate that the change from pre- to post-crisis was negative (positive) for decarbonisation. The year after the country name denotes the first year of crisis and the period below the country name denotes the entire analyses period. Time series figures for the GFC in Supplementary Note 7. Source:^31,32,57^.

However, previously improving trends in carbon or energy intensity can also worsen during and after crises. That was particularly notable in Greece during the GFC and Euro-crisis: the GDP fell substantially, temporarily reversing previous improvements in energy intensity. Still, as carbon intensity continues to decrease, the Greek economy is decarbonising, and emissions continued dropping after economic growth returned in 2017 (Fig. 4). These degrading effects are much more common in non-peak countries (Fig. 5), where the recovery tends to come with an increase in fossil fuel consumption and increasing energy intensity.

### The impact of crises in non-peak countries

In non-peak countries, GDP growth was less severely or not affected by the investigated crises, and the structural change effects lower than in peaking countries. GDP growth decreased by −1 (–2.4 to 0.1) percentage point in the post-crisis periods (*p* < 0.01), but remained positive thus increasing emissions; the structural change effect improved only by −0.2 (–2.2 to 1.3) percentage points, and this effect was not significant (Table 2). Consequently, emissions weakly decreased during the crises years but rebounded rapidly and kept increasing, triggered by a large positive GDP effect (generally growing both population and GDP per capita) outweighing a weak or emission-increasing structural change effect (Supplementary Notes 6, 7 and 8). Most countries in this group are emerging economies, where consumption of fossil fuels has increased rapidly in the last decades.

**Table 2:** Countries included in the analysis.

OECD members	OECD members (continued)	G20 but not OECD members
Austria	Korea (South)	*Argentina*
Australia	Latvia	*Brazil*
Belgium	Lithuania	*China*
Canada	Luxembourg	*India*
Chile	*Mexico*	*Indonesia*
*Colombia*	Netherlands	*Russia*
Czech Republic	New Zealand	*South Africa*
Denmark	Norway	Saudi Arabia
Estonia	Poland	
Finland	Portugal	
France	Slovakia	
Germany	Slovenia	
Greece	Spain	
Hungary	Sweden	
Iceland	Switzerland	
Ireland	*Turkey*	
Israel	United Kingdom	
Italy	United States	
Japan		

Countries in italic are low- and middle-income countries, all others are high-income countries, according to the World Bank classification in 2021^32^.

During the first and second oil crises, both the GDP and structural change effects explained rapid emissions increase in all non-peak countries (Supplementary Note 6). During the Soviet Union crisis, these countries were hardly economically affected, and GDP continued to increase emissions by more than 3% per year (median value) (Fig. 5). Because the contribution of structural change was positive or, in some cases, weakly negative, emissions kept rising in all these countries.

Again, non-peak countries were not hit hard by the GFC, and the GDP effect continued to increase emissions rapidly in the post-crisis period (Supplementary Note 7). The structural change effect deteriorated in some countries and improved in others, but in all non-peak countries it was too weak to compensate for the stronger GDP effect. Structural and GDP effects worsened after the GFC in Colombia and India, resulting in higher CO_2_ emissions rates. Australia, New Zealand and South Africa are exceptions: emissions slightly decreased in the post-GFC period, and these countries may have already peaked (Supplementary Note 2). Canada, Israel, and Mexico also show a level of structural change sufficient to compensate for economic growth, indicating that these countries are approaching the national peak.

## Discussion and conclusion

We have shown that peaks in CO_2_ emissions coincide with periods of economic crisis: of the 28 OECD and G20 countries that peaked emissions in the last 50 years, 26 did so just before or during an economic crisis resulting from geopolitical events or financial crashes. The peaks are explained by the combination of a lower GDP growth during and after the crisis and, importantly, by accelerated structural change, resulting in faster improvements in carbon and/or energy intensity post-crisis. After the peak, GDP continued to increase emissions in peaking countries, albeit at a lower level than before the crisis, making structural change improvements triggered during the recession or recovery the key post-crisis emission reduction driver. In all peak cases, the structural change effect reduced emissions post-crisis, and in 20 of 26 cases the effect strengthened or bent from positive to negative. This suggests that crises do not automatically trigger structural change, but they can be supportive, especially if work to improve energy and carbon intensity has already started. By contrast, non-peak countries were marginally or not affected by crises, and structural change effects were too weak to compensate for the strong GDP growth post-crisis, resulting in growing emissions.

This paper makes two main contributions to the ecological economics and climate policy literature. First, it extends the studies investigating drivers of declining CO_2_ emissions^27,28^ by explaining the impacts of all major economic crises on emissions peaks in OECD and G20 economies. Second, it adds to the scarce but growing ex-post evaluations of crises’ effects on decarbonisation^10,25^, by explicitly addressing the impacts on structural change. In line with conceptual work, it shows that crises have contributed to structural change in many major economies leading to CO_2_ emissions peaks. By analysing global trends only, previous studies may have thus underestimated the impacts of crises on decarbonisation.

Because national and even global economic and energy crises are recurrent phenomena, understanding their effects on decarbonisation is essential to design more resilient climate mitigation policies in the pathway to carbon neutrality by 2050. Most countries that have peaked did so not by “waiting for a crisis to come” but had already been implementing policies to improve energy efficiency and/or to develop less carbon-intensive energy: they were already improving energy or carbon intensity, or both, and this trend was strengthened in and following the crisis. The intensification of positive trends during crises suggests that some governments take advantage of times of economic instability to deepen support for policy reforms and “green Keynesianism” programs. This is a crucial difference between peaking and non-peaking countries, visible especially in the 1970s (as crisis-induced nuclear programmes intensified) and during the GFC (e.g. green recovery programmes in the US, Japan and EU). Also, in some cases, deep recessions -such as the ones in Poland and the Baltic states in the early 1990s or in Spain and Ireland during the GFC- destabilised entire economic sectors, favouring the deployment of new technologies and less emitting economic activities, highlighting the importance of creative-destruction mechanisms as suggested by previous studies^40,41^. Data on recovery packages during the Covid-19 pandemic^48^ indicate that countries that were already supporting the transition to a carbon-neutral energy system have expended the most on green sectors, taking the opportunity to strengthen their dominance in emerging zero carbon technologies and industries^16^.

Our findings also add to the green growth versus degrowth debate^49–52^: is degrowth necessary or desirable to reach a peak in emissions and eventually zero emissions? Our results show that absolute decoupling is possible: GDP continued to grow while domestic CO_2_ emissions decreased in the peak-and-decline group, but with important caveats. First, the rates of improvements in carbon and energy intensity rarely go below −4% per year, suggesting that GDP growth must be moderate, not surpassing a certain limit, if emissions are to be reduced. Second, reaching an absolute peak in emissions does not necessarily mean reaching zero emissions quickly: even the first economies to peak in the 1970s (e.g. Belgium, the UK and Germany) still have a long way to go to fully decarbonise their economies.

In line with previous research^25^, our findings do not mean that the effects of crises on decarbonisation are always positive. During the recovery period, countries can just build back the pre-crisis economy or step back to an even more carbon-intensive economy, such as the coal-based recovery in China and other countries after the Asian financial crisis of 1997^53^. In such cases, emissions do not peak post-crisis, but the emissions curve may even bend up. Policies supporting energy efficiency and clean energy must start before a crisis hits, so countries can have the opportunity to support already emerging cleaner industries during the recovery phase. Our findings do not mean that peaks will necessarily happen during recessions, as shown by the cases of Denmark and Switzerland (Supplementary Note 4), but they suggest that crises speed up the process making it possible to peak earlier.

Our approach has some limitations that call for more research. First, we worked with production- and not consumption-based emissions, as we investigate the effects of crises on national economies and their energy systems. Previous research has pointed to the transfer of emissions from developed to emerging economies as a potential driver of industrialised countries climate progress, although that effect seems to have stopped or clearly slowed down in the last 15 years^28,54^. Future research should address the possible impact of economic crises on demand and thus on consumption emissions. A second limitation refers to explanations of why emission peaks happened in some countries and not in others and the exact effects on peaking countries’ economic structure and energy systems. The observed structural change results from combinations of market forces and dedicated public policies, but the case-specific proportion was not investigated here. Further country-specific case study analysis would be required to answer questions about the root causes of each peak (and non-peak).

It is still too early to know which countries achieved a peak in carbon emissions during the Covid-19 pandemic and the current energy and economic crisis resulting from the war in Ukraine, and what their effects on structural, lasting change will be. The drop in GDP was very deep in the first half of 2020 but also short, with recovery starting quickly partly explained by a rapid policy response in all major economies through expansive fiscal and monetary policy. On the one hand, the short duration of the GDP fall suggests that this crisis’ creative-destruction effects could be limited. On the other hand, we also observe disruptions in global supply chains with impulse to re-localise production, which may alter previous globalisation trends and emission trajectories worldwide. Politically, the Covid-19 crisis also differs from previous ones. Since the signature of the Paris Agreement, there has been a growing consensus on the necessity to decarbonise the global economy as soon as possible. Therefore, many countries make their recovery packages green, explicitly seeking to build back better and use the crisis as leverage for green investments, thus helping accelerate technological change. This trend is strong in the industrialised countries that are already climate leaders, whereas climate laggard countries do not have green recovery packages or focus their recovery efforts on fossil fuel sectors^55^. Finally, the war in Ukraine has caused major disruption on the global energy system, similar in many respects to the oil crises in the 1970s. Again, in times of crisis, strategic decisions are being taken by governments and firms whose effects will be crucial for the objective of net-zero emissions before 2050.

## Methods

### Scope: countries investigated

Because this paper aims to explain when and how CO_2_ emissions peaks have occurred, and emissions peaks are more likely in industrialized economies^28^, our analysis includes all 37 OECD countries. It also includes 8 G20 countries that are not OECD members, thus comprising all major emitters except Iran. Together, this group of 45 countries (Table 2) accounted for 77% of global CO_2_ emissions in 2019^31^.

### Data sources

We base our CO_2_ and energy consumption data on statistics from BP^31^. The carbon emissions data reflect only emissions from combustion-related activities of oil, coal and natural gas and are based on ‘Default CO_2_ Emissions Factors for Combustion’ listed by the IPCC in its Guidelines for National Greenhouse Gas Inventories^56^. In this, we consider territorial emissions, but not ‘consumption-based’ emissions, because we are interested in the effect of crises on national economies—and one such effect could in principle be the outsourcing of emissions (e.g. shift of manufacturing industry abroad).

We use economic data -GDP in US Dollar (2015 constant)- and population data for 1965–2019 from the World Bank statistics^32^. GDP per capita data for countries that were part of the Soviet Union and Poland was taken from Maddison Project Database^57^. The GDP data for the post-Soviet sphere is thus different from the rest of our sample, but because our analysis is concerned with relative, not absolute, changes in GDP, this does not lead to an error or misleading comparison.

### Identification of CO_2_ emissions peaks

For each country, we first identified the year with the highest absolute value in CO_2_ emissions in 1965–2019. This metric may be biased by extreme weather conditions or other events that may have affected emissions in a particular year, but without being particularly relevant for the process of decarbonisation. To reduce the impact of such short-term fluctuations, we base our analysis on a 5-years moving mean (the unweighted mean of the previous 5 data points). Because emissions can temporarily decrease and then increase again, we apply a second condition for identifying countries with sustained emissions reductions: We identify a 5-year rolling average as peak only if it occurred at least ten years before the end of our data series in 2019. For potential peaks after 2009 it is still too early to know whether they were sustained (permanent) or temporary peaks. Our data indicate that Australia, New Zealand, Israel, and South Africa may have peaked after the GFC, but decreasing trends are not robust enough (Figure S3, Supplementary Note 7). Thus, we cannot be sure whether this is lasting or just a temporary dip in emissions, and we do not include these countries in the peak group.

We associate an economic crisis with an emission peak if it happens ± 2 years around the crisis’ onset. This way, we identify the peak-and-decline countries and especially the peak-and-decline countries where the peak is temporally connected to an economic crisis.

The method for identifying countries that achieved a peak in CO_2_ emissions used in this analysis is similar to the one applied by a previous paper^27^ that used a different dataset, coming out to a similar group of countries. That paper did not examine emission drivers or the influence of economic crises (Supplementary Note 1.4).

### Decomposition of CO_2_ emissions

This paper intends to show how CO_2_ emissions drivers were impacted by major economic crises in a large sample of countries. Studies on emissions drivers have applied several methods, including decomposition techniques and regression analysis, depending on the focus of each study. Because we focus on GDP and structural drivers, we apply Kaya-based decomposition of CO_2_ emissions, before and after each crisis and for each country. Kaya analysis^58^ is a common method applied across the climate mitigation literature^8^, which expresses emissions (tCO_2_) as a function of population (persons), GDP (2015 US$) and primary energy consumption (J), with the respective terms C, P, GDP and E:

1 $$C=P\left(\frac{{GDP}}{P}\right)\left(\frac{E}{{GDP}}\right)\left(\frac{C}{E}\right)$$

where GDP/P is GDP per capita (G), E/GDP in the energy intensity (EI) of GDP and C/E is the carbon intensity (CI) of energy.

We apply Index Decomposition Analysis (IDA) based on aggregate information at the country level, commonly used to perform cross-country comparisons and Kaya time-series analysis^59,60^. There are two variants of IDA: additive decomposition and multiplicative decomposition. In additive decomposition analysis, the arithmetic change of an aggregate indicator such as total CO_2_ emissions is decomposed, while in multiplicative decomposition the ratio change of an aggregate indicator is decomposed^60^. The multiplicative approach is more adequate for studies comparing different periods and countries^8^. Here, we implement multiplicative decomposition based on the Kaya identity and LMDI techniques proposed by Ang^60^, applied to national CO_2_ emissions, resulting in:

2 $$\varDelta C=\,\frac{{C}^{t}}{{C}^{0}}=\triangle P* \triangle G* \triangle {EI}* \triangle {CI}$$

3 $$\triangle P=\frac{{P}^{{{{{{\rm{t}}}}}}}}{{P}^{0}}$$

4 $$\triangle G=\frac{{G}^{t}}{{G}^{0}}$$

5 $$\triangle {EI}=\frac{{{EI}}^{t}}{{{EI}}^{0}}$$

6 $$\triangle {CI}=\frac{{{CI}}^{t}}{{{CI}}^{0}}$$

To estimate the contribution to emissions of the four Kaya factors in the periods before and after each crisis associated with an emissions peak, we assess trends ten years before the first year and after the last year of the crisis, defining the crisis as years with negative GDP growth. As the main variable of our interest is technological and structural change which takes time to materialize and to have an impact on emissions, if any, we study a relatively long period of 10 years. For example, for the Global Financial Crisis, the pre-crisis period is 1998–2007, as the crisis started in 2007 although its impacts on GDP manifested in 2008–09 in most countries; the post-crisis period ends in 2019, the last year of our analysis. The 10-year period is shorter when data is unavailable or when the post-crisis period overlaps with the next crisis, as occurred between the first (1973–75) and second (1979–80) oil crises. We derive the growth rates of emissions trends and Kaya factors over the pre- and post-crisis periods as follows:

7 $$r={\left(\frac{K\left(t+n\right)}{K\left(t\right)}\right)}^{1/n}-1$$

where *K* is the emissions value or Kaya factor in year *t*.

In this decomposition analysis we call the combined contribution of GDP per capita and population on CO_2_ emissions “GDP effect” and the combined contribution of carbon intensity and energy intensity “structural change effect”. Carbon intensity captures decarbonisation of energy supply systems, for example, fuel switching within fossil fuels (e.g., coal to gas) or switching from fossil fuels to renewables or nuclear. Economy-wide energy intensity represents changes that reduce the energy used per unit of GDP, such as energy conservation, increased energy performance of technologies, changes in the economic structure, and development of more efficient urban infrastructure. The impacts of energy and climate policy are reflected in the changes of carbon and energy intensities^6^ but not further investigated here.

### Statistical tests on GDP effect and structural change effect

We test whether differences in GDP and structural change (SC) effects before and after crises are statistically significant in peak and non-peak countries as in the whole sample. We test the following hypotheses:

1. Crises show a GDP effect for peak countries (i.e., the contribution factor decreased after crisis).
2. Crises show a SC effect for peak countries.
3. Crises show a SC effect for non-peak countries.
4. Crises show a GDP effect for non-peak countries.
5. Crises show a GDP effect for all countries.
6. Crises show a SC effect for all countries.

For doing so, we compare the growth rates of GDP and SC before and after the peak-related crises in peak-and-decline countries, all crises in non-peak countries, and all countries during all crises studied. Because the data are not normally distributed, we do not apply *t*-tests, but Wilcoxon signed-rank tests.

## Supplementary information

Supplementary Information

## Data Availability

The datasets analysed during the current study are available in the ZENODO repository: 10.5281/zenodo.7474121.

## Code availability

The codes generated during the current study are available in the ZENODO repository: 10.5281/zenodo.7477485.
