# Supplementary material for: Most industrialised countries have peaked carbon dioxide emissions during economic crises through strengthened structural change
Source: Commun Earth Environ. 2023 Feb 21;4(1):44. doi: 10.1038/s43247-023-00687-8 (PMC9942058; doi:10.1038/s43247-023-00687-8)
Supplement: Supplementary file 1 — Supplementary Information [file 43247_2023_687_MOESM1_ESM.pdf]

Supplementary material for:

Most industrialised countries have peaked carbon dioxide emissions during economic crises through strengthened structural change.

Germán Bersalli[1,*], Tim Tröndle[2], Johan Lilliestam[1,3]

[1] Energy Transitions & Public Policy group, Research Institute for Sustainability – Helmholtz Centre Potsdam, Potsdam, Germany.
[2] Climate Policy Lab, Institute for Environmental Decisions, ETH Zürich, Switzerland.
[3] Faculty of Economics and Social Sciences, University of Potsdam, Germany

[*] Corresponding author: german.bersalli@iass-potsdam.de

Supplementary Note 1: Literature review

1.1 Structural change and decarbonisation

The processes of economic growth, structural change, and anthropogenic greenhouse gas (GHG) emissions are intertwined, with structural change occurring at different levels (a region, a country, etc.) and dimensions. In a broad sense, structural change is understood as a set of interrelated changes in various dimensions of the economy, such as the sector compositions of output and employment, the organisation of industry, the financial system, income and wealth distribution, demography, and political and social institutions [1]. In the context of the energy transition, **structural change comprises shifts in the economic structure and the technologies used, particularly energy technologies, due to economic forces or public policy. These changes directly or indirectly affect energy intensity (unit of energy per unit of economic activity, usually GDP) and/or carbon intensity (unit of GHG emissions per unit of energy), and thus the level of emission.**

Various dimensions of structural change affect sustainability transitions and, particularly, the process of decarbonisation. In the most compressive review of the empirical evidence on that topic, [2] highlighted several domains of structural change as particularly important because of their impacts on climate change mitigation.

- **Economic sectors**: changes to the sectoral composition of the economy related, mostly, to shifts from manufacturing to services (tertiarisation) or from agriculture to manufacturing and changes between subsectors (e.g., from heavy to light industries). The sectoral composition of an economy influences different aspects of decarbonisation through input/output relations, which differ in terms of technical coefficients, labour coefficients, and energy intensity. Thus, different economic sectors contribute differently to GHG emissions. They also differ in terms of abatement costs and in innovative activity.

- **Industrial organisation**: changes in the organisation of production, through national and international value chains, have several effects on climate mitigation. First, increased specialisation is usually associated with higher productivity, which may influence energy intensity and innovation capacity. Second, changes in the organisation of production (for ex. firms' decision to make or buy) are also likely to modify the geographic concentration of economic activities and workers, an important factor in the contribution of transportation to GHG emissions. Third, outsourcing may come with increased international trade, which may imply offshoring more polluting activities from some countries to others.

- **Technical change**: it is central to both structural and climate change and includes changes to the energy mix, increased energy efficiency due to technical progress, and development and diffusion of less carbon-intensive goods and services. Innovations in zero-carbon technologies and energy efficiency technologies are crucial in the process of complete decarbonisation. It is important to distinguish between incremental innovations, which improve existing practices and products, and radical

innovations, which require significant changes in the economy, including consumer behaviour, the production structure, infrastructure, and related institutions.

- **Demand**: there are at least three aspects on the demand side of structural change that are critical to decarbonisation. First, income levels (e.g., GDP per capita) affect consumption patterns. Second, income distribution shapes the level of final consumption, the distribution of consumption across products, aversion to pollution and a country’s capacity to generate innovations. Third, consumers´ preferences change with income and over time. Preferences have been discussed extensively with respect to time: the risk aversion and the rate at which individuals discount future generations' consumption and costs of climate change´s impacts relative to present consumption and costs to reduce emissions.

- **Employment***:* it includes the need for employment relocations across industries and geography, as new industries demand new skills and novel combinations of know-how. The pace of adaptation of the workforce to new industries, geographies, technologies and tasks may induce a more or less efficient transition towards green technologies or impede it.

- **Institution***s*: Several aspects of structural change discussed here are influenced by institutions, including energy and climate governance, agency and power, social norms, and the organisation of critical industries for decarbonisation (e.g. electricity markets).

Some dimensions of structural change represent changes in national economies and others in the international configuration of the economic system -including global supply chains- and influence mutually. The empirical literature uses several variables to investigate the environmental impact of the above-mentioned production and consumption changes [1]. The most common include **energy intensity**, which is the ratio of energy inputs to GDP, and is inversely related to energy efficiency, and **carbon intensity** of the energy mix, which is related to the share of different fossil fuels, renewables and nuclear and measures the GHG content of energy consumption. In the empirical part of this paper, we use the concepts of energy and carbon intensities as indirect indicators of structural change; we do not investigate specific dimensions such as employment and institutions.

1.2 Theoretical expected effects of crises on structural change and decarbonisation

Environmental and ecological economists, political scientists, and scholars from other disciplines related to transition studies have discussed this question. Overall, they have pointed out several reasons why crises would positively impact climate mitigation and the transition to a carbon-neutral economy but also reasons to expect the opposite adverse effects.

In Schumpeterian economics, some scholars have linked the concept of evolutionary processes of creative destruction with economic crises. Mensch and Schnopp [3] argued that most of the “disequilibrium” trends, and specifically the shifts in trend, which have been observed during and since the Industrial Revolution, can be traced to changes in the rate and direction of technological innovation.

According to Mensh's analysis, in periods of crisis, the socioeconomic systems become structurally ready for a new spurt of basic innovation leading to a new (and different) cycle of growth. Major innovations would tend to cluster in periods of recessions because crises induce firms to examine drastically different technological options [3]. Also, crises would accelerate the decline of old and usually less efficient economic industries and support the emergence of new and more efficient ones. Authors in this stream [4] tend to consider deep crises as normal stages between the long-wave dynamics of techno-economic paradigm shifts: crises may form the tipping point towards the next (green) technological wave.

More recently, the question was examined by scholars related to "green Keynesianism" [5], focusing on reorienting fiscal and monetary policies and, more generally, on the green growth paradigm. It refers to the idea of reviving economic growth while resolving the problems of environmental decline and social injustice. The main purpose of green Keynesianism is, therefore, to recall the state for an active macroeconomic policy to tackle economic malaise and ecologic damage, channelling public spending toward low-carbon industries and environmentally friendly activities [6]. This view frames environmental protection as opportunity and reward rather than punishment or additional costs and looks for strategies to align economic growth and the environment. In the aftermath of an economic crisis, green growth can focus on creating new jobs in low-carbon sectors through public spending on green infrastructure and technologies -Keynesian green stimulus-, which stimulates aggregate demand. Thus, given the long lifetime of most energy infrastructures and technologies, countries should not miss the opportunities provided by crises to replace carbon-intensive technologies by cleaner alternatives. Modelling exercises suggest that the design of recovery packages and related choices between brown or green investment in the aftermath of the COVID-19 crisis may be critical for achieving the long-term climate targets [7].

Related to the concept of "critical junctures" from historical institutionalism in political science [8], crises can open up opportunities for new institutional pathways if the forces they unleash give rise to changes in existing norms, regulations and institutions. While institutional and policy processes are path-dependent and 'locked' into a certain policy pathway characterised by self-reinforcing feedback effects, an exogenous shock or crisis may trigger a shift away from existing paths toward new trajectories [9]. In fact, given the permanent competition for scarce resources, economic downturns should strengthen the case for a suitable design of climate policies which lead to cost-effective emissions reductions from an intertemporal perspective. Proponents of this view then call for clear, long-term, and stable policy frameworks and more international cooperation.

However, scholars have also highlighted potential negative effects of crises on the process of decarbonisation. By making access to capital more difficult, economic recessions may hinder emissions reduction efforts through their discouraging effects on investments in general, including investments in low- or zero-carbon technologies [10]. Moreover, lower energy prices in times of crisis, may reduce the

economic viability of cleaner technologies [11]. Political priorities may also shift again to decarbonisation: as both governments and the private sector focus on the recovery and on adapting their respective budgets, they may shift priorities away from climate policies. In this sense, crises tend to lead to deferment and postponement of environmental projects and investment as surviving the crisis and recovering becomes the aim, rather than becoming a “green” company or economy. Indeed, governments are likely to avoid burdening businesses and industries with extra costs and regulations when the economy is fragile and jobs may be at risk [11]. On the consumers´ side, lower incomes may encourage the consumption of goods with an inferior environmental quality (and lower prices). Thus, weaker environmental policies, reduced economic capacity for investments and depressed demand for greener products during crises may intensify carbon lock-in. This assumes, nonetheless, a low political will to implement climate policy in the short term, which may not be the case in many countries.

Transition studies scholars have also examined the impacts of crises on sustainability transitions [12-14]. Geels [13] highlighted the difficulty of the topic because crises are, by definition, confusing and contested phenomena which challenge existing ways of doing and understanding. Indeed, crises can disrupt existing institutions and cause uncertainty about future directions, which offers opportunities for substantial change that deviates from locked-in trajectories. Whether or not these opportunities are taken depends on how crises are interpreted (the dominant narrative) and the policy responses. In terms of the well-known multi-level perspective [15], crises can be seen as a shock at the landscape level. This shock creates pressures on regimes in concrete empirical domains (mobility, energy, etc.), where it may affect investor behaviour, availability of capital, public concerns, and the political will to act in favour of sustainability. At the niche level, many green innovations struggle against existing regimes. The broader diffusion of these niche innovations may require changes in the socio-technical regime: shifts in consumer practices, changes in public policies to favour green options, reorientations of incumbent firms and investors, and changes in public discourse [13]. Some or all these changes can emerge or intensify during periods of crisis.

1.3 Empirical evaluations of crises effects on decarbonisation

The empirical evidence on the effect of crises on CO_2 emissions and emissions drivers is scarce and offers conflictive results. Ex-post evaluations suggest that the short-term effects (1-2 years) of crises could be substantial [16, 17], but global emissions quickly bounce back, often overcompensating the crisis-related decrease. For example, Peters, Marland [18] found that the impact of the 2008–2009 global financial crisis on emissions was short-lived owing to strong emissions growth in emerging economies and a return to emissions growth in developed economies in 2010. The first studies of the Covid-19 crisis have pointed out that its effects on CO_2 emissions are likely to be only temporary because this crisis has not affected the fossil fuel-based energy system architecture [7, 19, 20]. Siddiqi [21] argued that the Asian financial crisis in 1998 reduced energy consumption temporarily but delayed plans to

invest in cleaner energy sources in affected countries. These analyses suggest that economic crises have no (positive) long-standing effects on decarbonisation.

However, other studies pointed to lasting effects. Using a sample of 68 countries for the period 1960 to 2014, Alsamara, Mimouni [22] found that the GFC has had permanent effects on CO_2 emissions and that these effects vary by countries` income level and depending on the severity of the crisis at the national level. Jalles [11] investigated a sample of 31 advanced and 55 emerging and low-income countries between 1980 and 2012. Their results suggest that financial crises, in general, led to a statistically significant fall in CO_2 and methane emissions; however, the effects can vary depending on the type of crisis (e.g., banking, currency or debt crisis) and on the monetary and fiscal situation before the crisis hits. By studying the effects of the collapse of the Soviet Union on energy intensities, Ürge-Vorsatz, Miladinova [23] found that energy sector and economic restructuring in the 1990s were very important in bringing down the high-energy intensities and carbon emissions in former communist countries in Easter Europe, particularly Poland. Similar results were found for a sample of 15 post-soviet republics [24]. Sobrino and Monzon [25] showed that the global financial crisis represented a turning point for Spanish CO_2 emissions from the transport sector due to a reduction in road transport and improved energy efficiency.

In sum, ex-post evaluations of crises effects are inconclusive and have not systematically investigated the effects of crises on structural change, which is paramount for decarbonisation in the long run.

1.4 Studies on CO_2 emission trends and drivers

The interlink between energy, economic growth and environmental degradation has been investigated through a variety of methods and approaches [26-28], including econometrics and decomposition analysis. Index Decomposition Analysis (IDA) has been widely used since the 1980s in studies dealing with drivers behind changes in energy consumption or energy intensity of a particular sector. Since 1990, IDA has been extended to study GHG emissions drivers, particularly energy-related CO_2 emissions. Xu and Ang [29] reviewed 80 IDA-based articles published from 1991 to 2012. These articles cover a wide range of developing and advanced economies and have been conducted for all major emissions sectors -electricity, industry, transport, and residential- including economy-wide studies. The review shows that energy intensity change was generally the critical driver of changes in aggregate carbon intensity in most sectors and countries. Taking energy intensity as a proxy for energy efficiency, this means improvements in energy efficiency have been the main driver of decreases in aggregate carbon emission. In contrast, the contributions of "activity structure" change and that of "carbon factor" change have been less significant. Besides, in all IDA studies, increases in overall activity levels invariably led to increases in emissions. Some different patterns appeared when comparing specific sectors and developing versus developed countries. Methodologically, different decomposition

techniques have been applied with diverse complexity; global studies use the simplest techniques, while country or sector-specific studies usually apply more detailed decomposition methods [29].

More recently, a growing number of IDA-based studies have focused on China, the global higher CO_2 emitter, in sectors like transport [30, 31], industry [32], residential [33], and electricity [34-37]. Analyses show that reduction rates in CO_2 emissions intensity markedly accelerated after 2013, the year when China´s Clean Air Action regulation was implemented; the authors suggest that pollution regulations on power plants and industries have been the most effective mitigation measures [38]. Research also highlights spatial heterogeneity among CO_2 dynamics in different Chinese provinces [33, 39, 40]; for example, while the western regions performed better in clean power penetration, the eastern regions performed better in thermal generation efficiency [34]. Outside China, IDA studies focused on countries in Latin America [41-43] and Europe [44, 45].

A recently published article reviews trends and drivers of GHG emissions in ten global regions and five economic sectors from 1990 to 2018 [46]. Overall, they show a moderate decarbonisation trend of energy systems in Europe and North America, driven by fuel switching and the increasing penetration of renewables. By contrast, fossil-based energy systems have continuously expanded in rapidly industrialising regions, only very recently slowing down in their growth. Papers focusing on global trends assessed Global CO_2 Emission Inequality through differences between production-based and consumption-based emissions [47] and the role of international trade in emissions [48] but paid little attention to the impact of crises. Apart from some crisis-specific or country-specific studies, no article assesses globally the role of crises on structural change and CO_2 emissions peaks, which is the aim of our article.

A few papers have analysed countries that have peaked emissions (Table S1). Using data from the Emissions Database for Global Atmospheric Research (EDGAR) v5 database, Lamb, Grubb [49] identified 24 countries showing sustained reductions in annual CO_2 and GHG emissions between 1970 and 2018, in total equalling 3.2 GtCO2eq since their respective emissions peaks. They found three groups of countries with different emissions pathways: six former eastern bloc countries, where emissions declined rapidly in the 1990s and have continued a downward trajectory since; six long-term decline countries, which have sustained reductions since the 1970s; and 12 recent peak countries, whose emissions decline began in the 2000s; In all cases, emissions reductions were achieved primarily in the energy systems sector. Most countries achieved emissions reductions alongside sustained economic growth, and some approached the fast annual rates of change that will be needed across the world in the coming decades to limit warming to 2°C. This paper did not investigate drivers leading to such GHG and CO_2 emissions peaks. Le Quéré, Korsbakken [50] examined drivers of declining CO_2 emissions in 18 developed economies. They show that, within this group, the partial displacement of fossil fuels by renewable energy and decreases in energy use explain decreasing CO_2 emissions. Besides, renewable

energy policies in these 18 countries have supported emissions reductions. These papers did not examine the impacts of economic crises on such emission peaks.

Table S1. Ex-post evaluations of crises´ short- and long-term effects on decarbonisation (top) – Studies on CO_2 emissions peaks (bottom).

Nr.	Reference	Scale	Crises & time span	Method	Main finding
**Crises ´ effects on emissions**					
1.	*This study*	*OECD + G20*	*All major crises, 1965-2019*	*Decomposition analysis*	*In 26 of 28 countries that have peaked CO_2 emissions, the peak occurred just before or during a major crisis, by the combined effect of lower GDP growth and decreasing energy and/or carbon intensity during and after the crisis;* ***In peak countries, crises have typically magnified pre-existing improvements in structural change***
2	Shammugam, Schleich [17]	Germany: CO_2 emissions, total and per sectors	Covid-19, 2020-21.	Decomposition analysis and autoregressive econometric models	About 58% of the reduction in emissions between 2019 and 2020 in Germany may be attributed to the pandemic. The buildings sector failed to meet its climate target in 2020
3	Ray, Singh [20]	184 countries	Covid-19, 2016-2020	Satellite based annual emissions observations; Spatial Analyst-Zonal Statistics tool	The total CO_2 emissions of selected 184 countries decreased by 438 Mt in 2020. Since global economic activities are expected to return to the non-COVID-19 state, the reduction in emissions during the pandemic will not be sustainable in the long run
4	Alsamara, Mimouni [22]	69 countries	GFC, 1960-2014	Time series regression analysis.	The GFC may have had a substantial influence on the relationship between economic growth and emissions. The CO_2 emissions and GDP per capita relationship follows an inverted U-shaped, and its turning point has shifted in response to the crisis.

6	Le Quéré, Peters [19]	Global	Covid-19, 1990-2020	Estimations based on the “confinement index during the COVID-19”	The pervasive disruptions from the COVID-19 pandemic have strongly affected CO_2 emissions in the short term: -2.6GtCO_2 in 2020 below 2019 (-7%)
7	Andreoni [16]	23 European countries	Covid-19, 2020 (first half)	Estimation based on GDP variations and sectoral carbon intensities	Short-term CO_2 reductions: 195,600 thousand tons of CO_2 have been avoided in January-June 2020, compared to the same period of 2019 (−12.1%)
8	Forster, Forster [7]	Global	Covid-19, 2020 (first half)	Emission-trend analysis, based on mobility data	The direct effect of the pandemic-driven response will be negligible, with a cooling of around 0.01 ± 0.005 ° C by 2030 compared to a baseline scenario that follows current national policies. In contrast, with an economic recovery tilted towards green stimulus and reductions in fossil fuel investments, it is possible to avoid future warming of 0.3 ° C by 2050. Without underlying long-term system-wide decarbonization of economies, even massive shifts in behaviour, only lead to modest reductions in the rate of warming
9	Sadorsky [51]	G20 countries	GFC, 2000-2017	Decomposition analysis	G20 countries as a whole showed lower CO_2 increases in the post-financial crisis period. China was the only exception.
10	Jalles [11]	86 countries	All financial crises, 1980-2012	Local projection method - impulse-response functions	Financial (banking, currency or debt) crises in general led to a statistically significant fall in CO_2 and methane emissions
11	Roinioti and Koroneos [52]	Greece	GFC, euro crisis, 2003-2013	Complete decomposition technique (Sun, 1998)	The reduction of CO2 emissions in Greece observed during the entire period examined and particularly during the crisis, was primarily the result of energy intensity improvement

12	Kopidou and Diakoulaki [53]	Greece, Italy, Portugal, *Spain:* industrial CO_2	GFC, 2000-11	Decomposition analysis	Production-based factors (mainly economic activity and energy intensity) contributed more to the change in industrial CO_2 emissions than the consumption-based factors. The consumers 'preferences did not switch to a more environmentally conscious use of industrial products
13	Timma, Zoss [54]	Latvia	GFC, 1996-2012	Decomposition analysis, mean-rate-of-exchange index	During the economic downturn of 2008-2010, GDP fell faster than energy consumption, resulting in increased energy intensity. This may be attributed to the fact that technological devices during the economic downturn did not work on full load; thus, the specific energy use and energy intensity indices increased
14	Sobrino and Monzon [25]	Spain – emissions from road transport	GFC, 1990-2010	Decomposition analysis	Spanish road traffic emissions decreased for the first time during the GFC. The reduction of road transport and improved energy efficiency has been powerful contributors to this decrease, demonstrating the effectiveness of energy-saving measures
15	Brizga, Feng [24]	15 post-Soviet republics	Soviet Union collapse, 1971-2010	Decomposition analysis	Overall, CO_2 emissions decreased sharply during the recession 1991-1997 and did not bond back during the following economic recovery, explained by economic restructuring
16	Peters, Marland [18]	Global	GFC, 2008-2010	CO_2 emissions estimations	The impact of the 2008–2009 global financial crisis (GFC) on emissions has been short-lived owing to strong emissions growth in emerging economies, a return to emissions growth in developed economies, and an increase in the fossil-fuel intensity of the world economy

17	Ürge-Vorsatz, Miladinova [23]	Poland, Hungary, Czech Republic	Soviet Union collapse, 1989-2002	Estimations of energy intensities	Energy sector and economic restructuring in the 1990s was very important in bringing down the high-energy intensities and carbon emissions in former communist countries in Easter Europe, particularly Poland
18	Siddiqi [21]	South-East Asia	Asian financial crisis, 1998	Unspecified	The crisis had short-term positive effects on decreasing energy consumption and CO_2 emissions but may has delayed plans to invest in cleaner energy sources
**CO_2 emissions peaks**					
1	Lamb, Grubb [49]	24 countries	1970-2018	Descriptive statistics	24 countries have sustained CO_2 and GHG emissions reductions between 1970 and 2018. Most countries achieved emissions reductions alongside sustained economic growth, and some approached the fast annual rates of change that will be needed across the world in the coming decades to limit warming to 2° C but not to 1.5° C,
2	Le Quéré, Korsbakken [50]	18 developed economies	2005-2015	LMDI decomposition	The displacement of fossil fuels by renewable energy and decreases in energy use, both supported by public policies, explain decreasing CO_2 emissions in 18 peak-and-decline countries. However, the decrease in energy use can be explained at least in part by a lower growth in gross domestic product.

Supplementary Note 2: Temporality of CO_2 emissions peaks

From our sample of 45 countries, we found 28 countries that have peaked CO_2 emissions and 17 that have not, according to the highest value in the CO_2 emissions series applying a 5-year moving average (Table S2). The peak timing shows that three countries peaked during the First Oil Crisis, four during the Second Oil Crisis, six during the economic crisis related to the collapse of the Soviet Union, and 12 countries during the Global Financial Crisis. It means that emissions reached a maximum point before the crisis, decreased substantially during it, and never reached the pre-crisis levels again. Results suggest that New Zealand reached a peak in 2008; however, observing the data closely, we see that emissions have followed an upward trend after 2011; thus, we cannot be sure whether 2008 was the maximum (permanent) peak or a temporal one. A similar situation occurred in Australia, Israel, and South Africa, where it is unclear if the peaks in 2011/12 were permanent. Thus, we decided to exclude these four countries from the peak-and-decline group. In sum, we found 26 countries which peaks related to an economic crisis, two countries where the peaks happened outside crises and 17 non-peak countries (including the four countries which may have peaked, but we are only going to know some years later).

Suppose that instead of taking a 5-year rolling average, we take the year of maximum CO_2 emissions - the highest absolute value in CO_2 emissions in 1965-2019. In that case, we arrive at the same peaks associated with the same crises in most cases, with some differences. For example, Finland, Austria and Norway may have peaked some years early, around 2003; Yet a clear decline after the GFC can be observed. On the contrary, Canada, Australia, and South Africa may have peaked during the GFC. However, as explained in the Method section, we prefer a 5-years rolling average method (as described above) to avoid the influence of a simple year with exceptionally high emissions, which not necessarily reflects the process of decarbonization.

Country	Peak year	Crisis	Country	Peak year	Crisis
BEL	1973	First Oil	AUS	2011	No peak
GBR	1973	First Oil	ISR	2012	No peak
LUX	1974	First Oil	ZAF	2012	No peak
SWE	1980	Second Oil	ARG	2017	No peak
FRA	1980	Second Oil	BRA	2017	No peak
DEU	1980	Second Oil	MEX	2017	No peak
CZE	1981	Second Oil	KOR	2019	No peak
HUN	1982	Second Oil	ISL	2019	No peak
SVK	1988	Soviet Union	CHL	2019	No peak
LVA	1989	Soviet Union	COL	2019	No peak
POL	1989	Soviet Union	CAN	2019	No peak
EST	1990	Soviet Union	SAU	2019	No peak
RUS	1990	Soviet Union	TUR	2021	No peak
LTU	1991	Soviet Union	CHN	2021	No peak
DNK	1998	No crisis	IDN	2021	No peak
CHE	2001	No crisis	IND	2021	No peak
PRT	2005	Global Financial			
FIN	2006	Global Financial			
AUT	2006	Global Financial			
NOR	2007	Global Financial			
ITA	2007	Global Financial			
USA	2007	Global Financial			
JPN	2008	Global Financial			
IRL	2008	Global Financial			
GRC	2008	Global Financial			
NLD	2008	Global Financial			
NZL	2008	Global Financial			
SVN	2008	Global Financial			
ESP	2008	Global Financial			

Table S2. Year of CO_2 peak – year with the higest 5-year moving average-. Source: [55]

Supplementary Note 3: Decoupling between GDP and CO_2 emissions

A decoupling analysis is a commonly used approach to understand whether a variable grows while another variable is increasing or decreasing. In environmental science, this is often done with GDP and emissions of some pollutant or consumption of a material; here, we investigate the coupling state of GDP and CO_2 emissions. Decoupling factors indicate the change in one unit of the environmental pressure, in our case CO_2 emissions, to the change of one unit of the economic driving force, here GDP, estimated at time *t* and compared with the base period *0*. It can be formalised as follows [56-58]:

$$DF = 1 - \frac{\frac{CO2(t)}{GDP(t)}}{\frac{CO2(0)}{GDP(0)}} \qquad (1)$$

A decoupling factor of zero (DF = 0) means the absence of any decoupling. A value of one (DF= 1) implies perfect decoupling such that carbon emissions are reduced to zero at time *t*. Negative values (DF < 0), by contrast, imply coupling. Using Eq. (1) we derived decoupling factors for all peak-and-decline countries, considering the period from the year of the CO_2 emissions peak to 2019.

Decoupling State	Condition	Growth paradigm
(1) Absolute decoupling	(-)ΔCO2 & (+)ΔGDP	Green growth
(2) Relative decoupling	(+)ΔCO2 < (+)ΔGDP	Low-carbon growth
(3) Expansive coupling	(+)ΔCO2 > (+)ΔGDP	Carbon-intensive growth
(4) Negative decoupling	(+)ΔCO2 & (-)ΔGDP	Dirty decline
(5) Negative coupling	(-)ΔCO2 & (-)ΔGDP	De-growth

Table S3: Identification of decoupling states

We distinguish between five decoupling states following the approach proposed by Naqvi and Zwickl [57] and use them to analyze changes in carbon emissions and economic growth dynamics in the countries studied. First, "absolute decoupling" requires that CO_2 emissions decrease while GDP grows, so $\Delta CO_2 \leq 0$ and $\Delta GDP > 0$. This corresponds with the "green growth" discourse, seeking to continue growth while reducing (and eventually eliminating) emissions. Second, "relative decoupling", occurs when both variables increase but the economy grows faster than the environmental bad [56], such that $\Delta GDP > \Delta CO_2$. This state corresponds to "low-carbon growth" and is, barring massive CO_2 removal schemes, incompatible with the net-zero emissions implication Paris Agreement. Third, "expansive coupling", occurs when both variables are positive but in this case, CO_2 emissions increase faster than economic output. We include two last states referencing the situation where economic activity decreases. Fourth, "negative decoupling" refers to the possible but unlikely situation in which economic growth is negative, but emissions increase. Fifth, “negative coupling” occurs when both CO_2 emissions and economic output decrease, referring to a “de-growth” situation. Only the first and fifth states mean

that the economy is decarbonizing. Negative coupling, however, may imply more severe social and political troubles and is still not considered a target by any government, although it is gaining attention in climate policy research [59].

Country	Peak	ΔCO_2	ΔGDP	DF	State
Belgium	1973	-17%	146%	0.66	Absolute decoupling
United Kingdom	1973	-48%	154%	0.80	Absolute decoupling
Luxemburg	1974	-30%	352%	0.84	Absolute decoupling
Sweden	1980	-53%	131%	0.80	Absolute decoupling
Germany	1980	-37%	94%	0.67	Absolute decoupling
France	1980	-38%	101%	0.69	Absolute decoupling
Czechia	1981	-48%	157%	0.80	Absolute decoupling
Hungary	1982	-42%	123%	0.74	Absolute decoupling
Slovakia	1988	-44%	122%	0.75	Absolute decoupling
Latvia	1989	-59%	12%	0.63	Absolute decoupling
Poland	1989	-30%	203%	0.77	Absolute decoupling
Estonia	1990	-41%	34%	0.56	Absolute decoupling
Russia	1990	-30%	94%	0.64	Absolute decoupling
Lithuania	1991	-68%	59%	0.80	Absolute decoupling
Denmark	1998	-50%	36%	0.63	Absolute decoupling
Switzerland	2001	-17%	40%	0.41	Absolute decoupling
Portugal	2005	-22%	10%	0.22	Absolute decoupling
Finland	2006	-37%	10%	0.43	Absolute decoupling
Austria	2006	-12%	18%	0.26	Absolute decoupling
Norway	2007	-6%	14%	0.18	Absolute decoupling
United States	2007	-15%	22%	0.31	Absolute decoupling
Italy	2007	-28%	-4%	0.25	Negative coupling
Netherlands	2008	-14%	11%	0.23	Absolute decoupling
Japan	2008	-13%	7%	0.19	Absolute decoupling
Ireland	2008	-22%	71%	0.54	Absolute decoupling
Spain	2008	-22%	7%	0.27	Absolute decoupling
Slovenia	2008	-17%	5%	0.21	Absolute decoupling
Greece	2008	-36%	-23%	0.17	Negative coupling

Table S4: **Decoupling factors (DF) and decoupling states in peak-and-decline countries;** ΔGDP and ΔCO_2 denote the increase of GDP and CO_2 emissions (in %) between the year of emissions peaks and 2019; Sources: [55, 60, 61].

The results (Table S4) show that all but two peak-and-decline countries experienced absolute decoupling in the period peak year-2019, meaning that CO_2 emissions reached a maximum and then decreased while GDP grew. Because the first countries peaked already in the early 1970s, absolute decoupling is possible even for long periods. However, the trajectories after the peak can vary substantially. For instance, the UK and Belgium both peaked in 1973, but whereas Belgium reduced

emissions by only 17% and increased GDP by 146% in 1973-2019, the UK reduced emissions by 48% despite stronger GDP growth. Notably, Ireland peaked emissions in 2008 but then reduced emissions faster than other countries, even with a booming economic activity, suggesting that substantial transformation has happened in the Irish economy and its energy system in the last decade.

The two exceptions in this group are Italy and particularly Greece, which have been in a state of negative coupling since the Global Financial Crisis. Both countries peaked emissions at the beginning of the crisis and then experienced deep recessions accompanied by a substantial reduction in emissions. The GDP started to grow again but had not yet reached pre-crisis levels in 2019.

Supplementary Note 4: CO_2 emission peaks in Denmark and Switzerland

Denmark (1998) and Switzerland (2001) are the only two peak-and-decline countries in our group where emissions peaked outside major economic crises. Switzerland had a minor national recession in 2002-03, and during the recovery years, the energy intensity decreased firmly, preventing emissions from increasing. There was no national economic crisis in Denmark around the emission peak year, and structural change alone explained the peak in emissions, due to substantial improvement in both energy and carbon intensities.

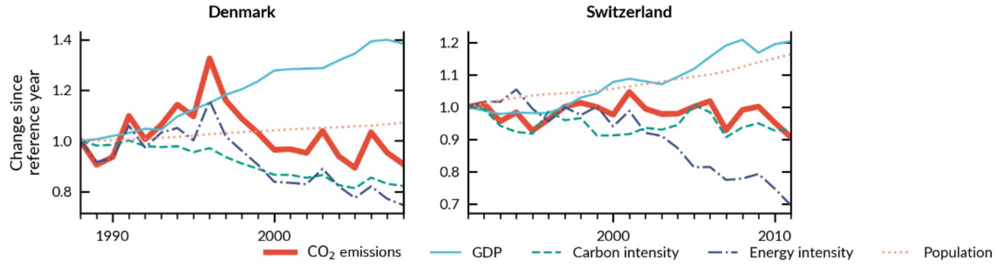

Figure S1: Emission driver decomposition in Denmark (1988-2007) and Switzerland (1991-2010). Sources:[55, 60].

Supplementary Note 5: Changes in the energy mix in peak-and-decline countries

A group of Western European countries were the first to peak CO_2 emissions during the first and second oil crises. These early peaks were associated with a decrease in oil consumption (Table S5) mainly due to two factors: first, the implementation of conservation and energy efficiency measures as a response to the crises; and second, the acceleration of nuclear power development (Table S6), to replace oil power generation. Additionally, some countries like Sweden accelerated the deployment of biofuels. RD&D in renewables for electricity generation like solar and wind increased, but the deployment of these technologies was very low, not affecting the energy mix in the 1970s.

In countries that peaked during the collapse of the Soviet Union, the fuel switch effect was less significant. These countries experienced a substantial drop in primary energy consumption from all sources, particularly oil (Table S7). When Russia and the Baltic countries recovered from the deep recession, oil consumption remained substantially lower than before the crisis, translating into energy intensity improvements.

The fuel switch in the countries that peaked during the Global Financial Crisis was different. Most of these countries saw a decrease in coal consumption during and after the crisis (Table S8). In the case of Spain, one of the countries hardest hit by the crisis, coal consumption fell by half between 2007 and 2009. Simultaneously, the deployment of renewable energy did not stop and even accelerated in some cases, such as in the US (Table S9). Even in countries that suffered strong recessions, such as Italy and Greece, the deployment of renewable energies continued. The decrease in coal consumption and the increase in renewables improved the carbon intensity in this group of countries. In parallel, oil consumption continued to decrease while gas consumption increased, contributing to improvements in carbon intensity.

Table S5: Oil consumption* in countries that peaked during the First Oil Crisis (EJ)**.** Numbers in red highlight the year of consumption peak.

	1965	1966	1967	1968	1969	1970	1971	1972	1973	1974	1975	1976	1977	1978	1979	1980	1981	1982	1983	1984
**Belgium**	0.68	0.69	0.76	0.89	1.02	1.12	1.14	1.24	1.30	1.14	1.09	1.13	1.11	1.19	1.18	1.09	1.00	0.95	0.86	0.83
**France**	2.29	2.46	2.83	3.06	3.54	4.02	4.38	4.86	5.42	5.15	4.71	5.09	4.89	5.10	5.07	4.71	4.25	3.94	3.85	3.71
**Germany**	3.67	4.11	4.27	4.79	5.39	5.92	6.17	6.51	6.94	6.29	6.11	6.61	6.52	6.81	7.02	6.35	5.76	5.45	5.34	5.32
**Luxembourg**	0.04	0.04	0.04	0.05	0.05	0.06	0.06	0.06	0.07	0.06	0.06	0.06	0.06	0.06	0.06	0.05	0.04	0.04	0.04	0.04
**Sweden**	0.83	0.93	0.93	1.05	1.13	1.23	1.15	1.19	1.21	1.09	1.09	1.20	1.16	1.40	1.51	1.33	1.20	1.11	0.97	0.92
**United Kingdom**	3.19	3.42	3.67	3.89	4.18	4.42	4.45	4.72	4.84	4.50	3.93	3.91	3.94	4.04	4.08	3.49	3.23	3.27	3.14	3.86

* Inland demand plus international aviation and marine bunkers and refinery fuel and loss. Consumption of biogasoline and biodiesel are excluded while derivatives of coal and natural gas are included.
Source: [62].

Table S6: Nuclear energy consumption* in countries that peaked during the First Oil Crisis (EJ, input-equivalent).

	1965	1966	1967	1968	1969	1970	1971	1972	1973	1974	1975	1976	1977	1978	1979	1980	1981	1982	1983	1984
**Belgium**	-	^	^	^	^	^	-	^	^	^	0.07	0.10	0.12	0.13	0.11	0.13	0.13	0.16	0.24	0.28
**France**	0.01	0.01	0.02	0.03	0.04	0.06	0.09	0.15	0.15	0.15	0.18	0.16	0.18	0.30	0.40	0.61	1.05	1.09	1.44	1.91
**Germany**	^	^	0.01	0.02	0.05	0.06	0.06	0.10	0.12	0.14	0.24	0.30	0.41	0.44	0.52	0.56	0.66	0.74	0.78	1.04
**Luxembourg**	-	-	-	-	-	-	-	-	-	-	-	-	-	-	-	-	-	-	-	-
**Sweden**	^	^	^	^	^	^	^	0.01	0.02	0.02	0.12	0.16	0.20	0.24	0.21	0.26	0.38	0.39	0.41	0.51
**United Kingdom**	0.15	0.20	0.23	0.26	0.29	0.26	0.28	0.29	0.28	0.34	0.30	0.36	0.40	0.37	0.38	0.37	0.38	0.44	0.50	0.54

* Based on gross generation and not accounting for cross-border electricity supply.

"Input-equivalent" energy is the amount of fuel that would be required by thermal power stations to generate the reported electricity output
^ Less than 0.005
Source: [62].

Table S7: Oil consumption* in countries that peaked during the Soviet Union crisis (EJ)**.** Numbers in red highlight the year of consumption peak.

	1985	1986	1987	1988	1989	1990	1991	1992	1993	1994	1995	1996	1997	1998	1999	2000	2001	2002	2003	2004
**Slovakia**	0.26	0.26	0.25	0.24	0.24	0.21	0.18	0.17	0.14	0.14	0.14	0.15	0.15	0.16	0.15	0.15	0.14	0.15	0.14	0.14
**Latvia**	0.24	0.21	0.17	0.15	0.15	0.14	0.14	0.10	0.09	0.09	0.08	0.08	0.07	0.07	0.07	0.05	0.06	0.06	0.06	0.07
**Poland**	0.70	0.72	0.73	0.75	0.75	0.68	0.65	0.59	0.61	0.64	0.65	0.76	0.79	0.90	0.94	0.87	0.85	0.86	0.88	0.94
**Estonia**	0.15	0.14	0.15	0.15	0.15	0.15	0.13	0.07	0.07	0.07	0.05	0.06	0.06	0.06	0.05	0.05	0.06	0.06	0.06	0.06
**Russia**	10.57	10.70	10.78	10.70	10.90	10.74	10.47	10.00	8.34	7.37	6.43	5.54	5.50	5.27	5.38	5.28	5.43	5.24	5.43	5.36
**Lithuania**	0.37	0.31	0.34	0.33	0.33	0.32	0.35	0.18	0.16	0.15	0.13	0.14	0.14	0.16	0.13	0.10	0.12	0.11	0.10	0.11

* Inland demand plus international aviation and marine bunkers and refinery fuel and loss. Consumption of biogasoline and biodiesel are excluded while derivatives of coal and natural gas are included.

Table S8: Coal consumption* in countries that peaked during the Global Financial Crisis (EJ). Numbers in red highlight the year of consumption peak

	1998	1999	2000	2001	2002	2003	2004	2005	2006	2007	2008	2009	2010	2011	2012	2013	2014	2015	2016	2017	2018	2019
**US**	21.71	21.67	22.63	21.96	21.95	22.37	22.52	22.85	22.50	22.80	22.44	19.74	20.88	19.70	17.42	18.08	18.04	15.58	14.26	13.87	13.28	11.34
**Japan**	3.54	3.73	4.00	4.09	4.35	4.47	4.59	4.81	4.72	4.96	5.09	4.27	4.87	4.62	4.88	5.07	4.99	5.03	5.02	5.10	4.99	4.91
**Austria**	0.14	0.14	0.15	0.16	0.16	0.17	0.17	0.17	0.17	0.16	0.16	0.12	0.14	0.15	0.13	0.14	0.13	0.14	0.13	0.13	0.12	0.12
**Finland**	0.23	0.22	0.21	0.25	0.28	0.34	0.31	0.20	0.31	0.29	0.22	0.22	0.28	0.23	0.19	0.21	0.19	0.16	0.18	0.17	0.18	0.15
**Greece**	0.37	0.36	0.38	0.39	0.38	0.38	0.39	0.38	0.35	0.37	0.35	0.35	0.33	0.33	0.34	0.29	0.28	0.24	0.18	0.20	0.19	0.22
**Ireland**	0.12	0.10	0.11	0.11	0.11	0.11	0.10	0.11	0.10	0.10	0.10	0.08	0.08	0.08	0.09	0.09	0.08	0.09	0.09	0.08	0.06	0.04
**Italy**	0.49	0.49	0.52	0.56	0.57	0.62	0.69	0.69	0.70	0.68	0.66	0.52	0.57	0.64	0.66	0.57	0.55	0.52	0.46	0.40	0.37	0.28
**Netherlands**	0.36	0.31	0.33	0.35	0.35	0.36	0.36	0.34	0.32	0.35	0.33	0.31	0.32	0.31	0.34	0.34	0.38	0.46	0.43	0.39	0.35	0.27
**Norway**	0.04	0.04	0.04	0.04	0.03	0.03	0.04	0.03	0.03	0.03	0.03	0.02	0.03	0.04	0.03	0.03	0.04	0.03	0.03	0.03	0.03	0.03
**Portugal**	0.15	0.16	0.16	0.13	0.15	0.14	0.14	0.14	0.14	0.12	0.11	0.12	0.07	0.09	0.12	0.11	0.11	0.14	0.12	0.14	0.11	0.05
**Slovenia**	0.06	0.05	0.06	0.06	0.07	0.06	0.06	0.06	0.06	0.07	0.06	0.06	0.06	0.06	0.06	0.06	0.04	0.04	0.05	0.05	0.05	0.04
**Spain**	0.73	0.82	0.88	0.80	0.90	0.84	0.88	0.86	0.75	0.84	0.57	0.39	0.29	0.54	0.65	0.48	0.49	0.57	0.44	0.56	0.46	0.16

* Commercial solid fuels only, i.e. bituminous coal and anthracite (hard coal), and lignite and brown (sub bituminous) coal, and other commercial solid fuels.
Excludes coal converted to liquid or gaseous fuels, but includes coal consumed in transformation processes.

Source: [62]

Table S9: Renewable energy consumption* in countries that peaked during the Global Financial Crisis (EJ, input-equivalent).

	1998	1999	2000	2001	2002	2003	2004	2005	2006	2007	2008	2009	2010	2011	2012	2013	2014	2015	2016	2017	2018	2019
**US**	0.80	0.83	0.86	0.88	0.99	1.05	1.14	1.22	1.44	1.65	2.06	2.34	2.70	3.03	3.27	3.70	3.98	4.19	4.74	5.17	5.44	5.71
**Japan**	0.16	0.17	0.17	0.17	0.18	0.19	0.20	0.24	0.25	0.26	0.26	0.25	0.29	0.30	0.33	0.39	0.49	0.64	0.67	0.80	0.90	1.01
**Austria**	0.02	0.02	0.02	0.02	0.02	0.02	0.03	0.04	0.06	0.07	0.08	0.08	0.08	0.08	0.09	0.10	0.11	0.12	0.12	0.13	0.13	0.15
**Finland**	0.09	0.08	0.09	0.08	0.09	0.09	0.10	0.09	0.11	0.10	0.10	0.09	0.11	0.12	0.12	0.13	0.14	0.14	0.14	0.17	0.18	0.19
**Greece**	^	^	^	0.01	0.01	0.01	0.01	0.01	0.02	0.02	0.03	0.03	0.03	0.04	0.06	0.08	0.08	0.09	0.09	0.10	0.10	0.12
**Ireland**	^	^	^	^	^	0.01	0.01	0.01	0.02	0.02	0.03	0.03	0.03	0.05	0.04	0.05	0.06	0.07	0.07	0.08	0.09	0.10
**Italy**	0.05	0.06	0.07	0.07	0.09	0.10	0.12	0.13	0.14	0.15	0.19	0.24	0.30	0.40	0.53	0.60	0.61	0.63	0.65	0.67	0.64	0.65
**Netherlands**	0.02	0.02	0.03	0.03	0.04	0.04	0.05	0.07	0.08	0.09	0.10	0.12	0.11	0.13	0.13	0.13	0.12	0.14	0.15	0.18	0.19	0.23
**Norway**	^	^	^	^	^	0.01	0.01	0.01	0.01	0.01	0.02	0.02	0.02	0.02	0.02	0.03	0.03	0.03	0.04	0.05	0.05	0.07
**Portugal**	0.01	0.01	0.02	0.02	0.02	0.02	0.02	0.03	0.05	0.06	0.08	0.10	0.13	0.13	0.14	0.16	0.16	0.16	0.16	0.16	0.16	0.18
**Slovenia**	-	^	^	^	^	^	^	^	^	^	^	^	^	^	0.01	0.01	0.01	0.01	0.01	0.01	0.01	0.01
**Spain**	0.03	0.04	0.07	0.09	0.12	0.16	0.19	0.24	0.26	0.30	0.38	0.46	0.57	0.59	0.70	0.72	0.69	0.67	0.66	0.68	0.69	0.73

*Includes renewable power (apart from hydro, which is reported separately) and biofuels

“Input-equivalent” energy is the amount of fuel that would be required by thermal power stations
^ Less than 0.005.
Source: [62]

Supplementary Note 6: CO_2 emission Kaya decomposition, all countries.

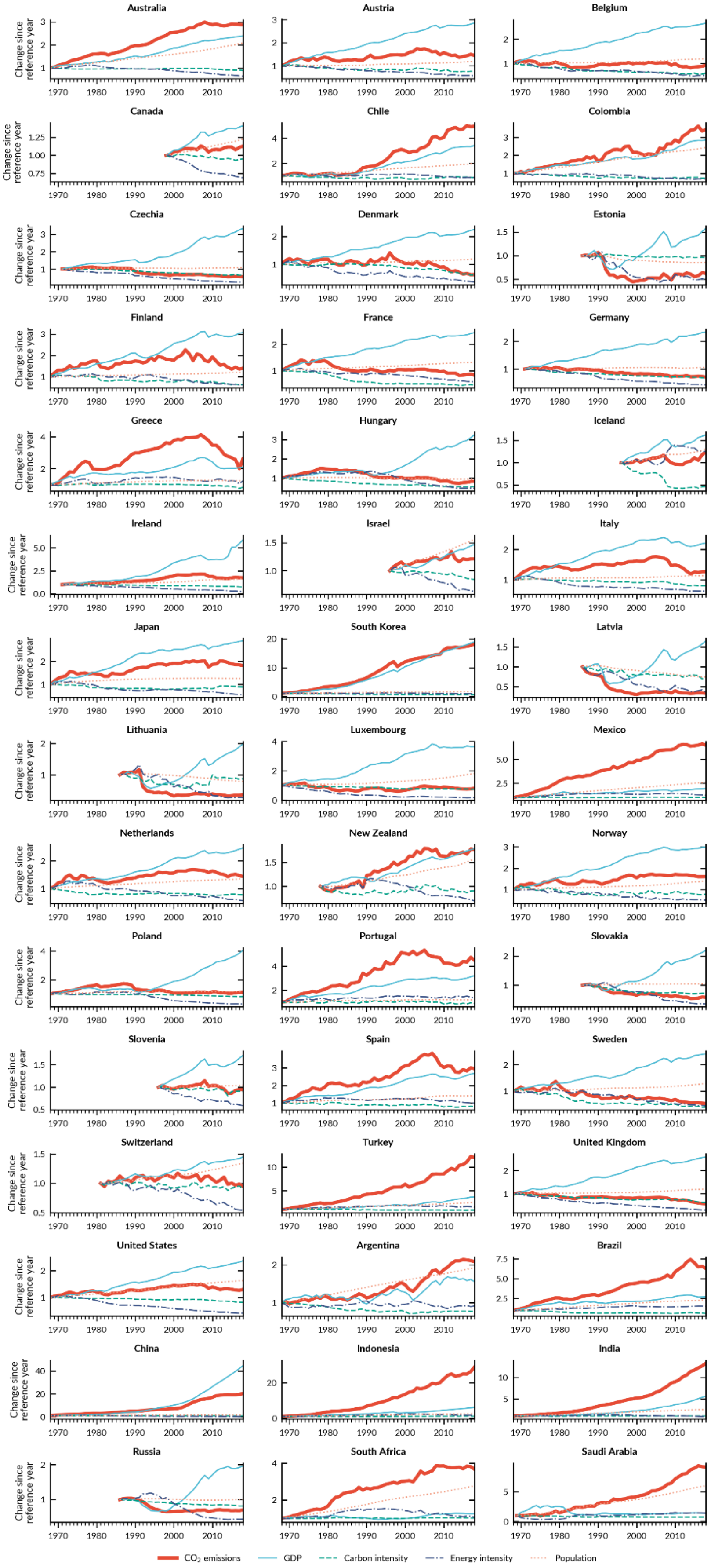

Figure S2: Kaya-factors decomposition - all countries, whole period. Sources: [55, 60, 61]

Supplementary Note 7: The impacts of the GFC crisis on non-peak countries. - CO_2 emission Kaya decomposition.

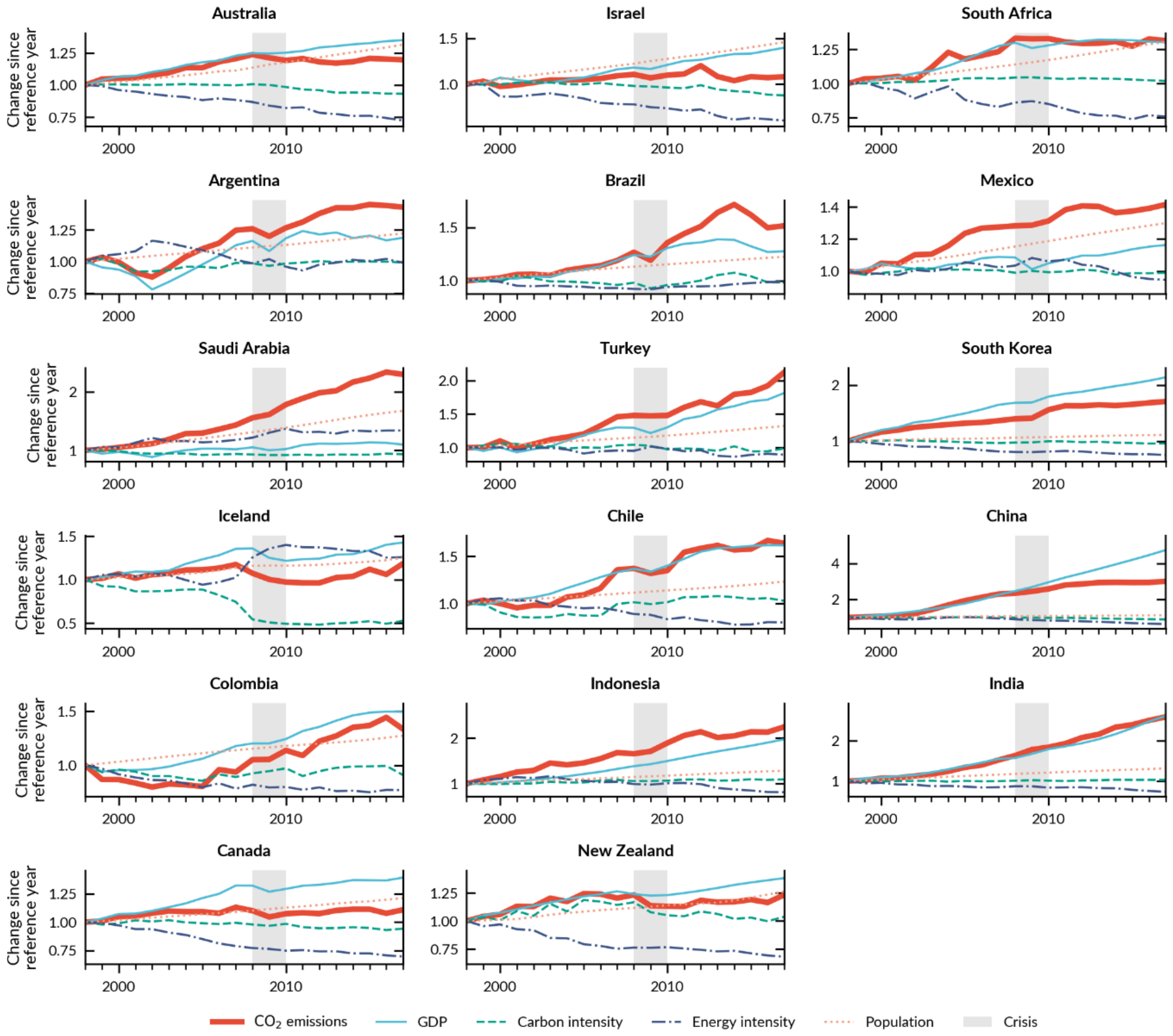

Figure S3: Emission driver decomposition in non-peak countries (1998-2019). Sources: [55, 60].

Supplementary Note 8: Kaya decomposition factors, all countries.

Table S10: Decomposition factors first oil crisis. Yearly average CO_2 emission and decomposition factors (GDP effect and Structural Change effect), for peak (left) and non-peak (right) countries before and after the first oil crisis (1974-75). Green (orange) indicates that the factor contribution change post-crisis was positive (negative) for decarbonisation. Sources:[55, 60].

	Pre-crisis			Post-crisis		
	GDP	SC	CO2	GDP	SC	CO2
BEL	4.9%	-2.7%	2.2%	1.9%	-1.7%	0.2%
GBR	4%	-3.2%	0.8%	2.3%	-2.6%	0.2%
LUX	5.4%	-3.6%	1.8%	0.7%	-5.1%	-4.4%
Median	*4.9%*	*-3.2%*	*1.8%*	*1.9%*	*-2.6%*	*0.2%*

	Pre-crisis			Post-crisis		
	GDP	SC	CO2	GDP	SC	CO2
ARG	3.7%	-1.8%	1.9%	2.0%	-0.2%	1.8%
AUS	4.8%	0.4%	5.2%	2.5%	0.9%	3.4%
BRA	9.7%	1.7%	11.4%	6.4%	-0.6%	5.8%
CHL	3.3%	-0.1%	3.2%	3.1%	-2.3%	0.9%
CHN	6.6%	2.4%	9.0%	6.6%	1.0%	7.6%
COL	6.0%	-2.1%	3.9%	5.0%	-1.8%	3.2%
IDN	6.4%	-1.3%	5.1%	7.0%	7.1%	14.1%
IND	3.4%	0.0%	3.4%	3.6%	0.8%	4.4%
KOR	11.4%	1.7%	13.1%	10.6%	0.9%	11.5%
MEX	6.4%	0.7%	7.1%	6.4%	1.8%	8.2%
TUR	5.8%	4.1%	9.9%	4.3%	0.5%	4.8%
ZAF	4.5%	-0.4%	4.1%	2.1%	1.1%	3.3%
Median	*5.9%*	*0.2%*	*5.1%*	*4.7%*	*0.9%*	*4.6%*

Table S11: Decomposition factors second oil crisis. Yearly average CO_2 emission and decomposition factors (GDP effect and Structural Change effect), for peak (left) and non-peak (right) countries before and after the second oil crisis (1980-81). Green (orange) indicates that the factor contribution change post-crisis was positive (negative) for decarbonisation. Sources:[55, 60].

	Pre-crisis			Post-crisis		
	GDP	SC	CO2	GDP	SC	CO2
CZE	2.6%	-2.4%	0.3%	1.6%	-4.0%	-2.4%
DEU	3.5%	-3.1%	0.4%	2.5%	-3.7%	-1.2%
FRA	3.7%	-3.5%	0.2%	2.3%	-4.2%	-1.8%
HUN	4.3%	1.6%	5.9%	1.0%	-2.3%	-1.3%
SWE	1.3%	5.8%	7.1%	2.2%	-6.0%	-3.8%
Median	*3.5%*	*-2.4%*	*0.4%*	*2.2%*	*-4.0%*	*-1.8%*

	Pre-crisis			Post-crisis		
	GDP	SC	CO2	GDP	SC	CO2
ARG	4.0%	-1.2%	3.3%	0.1%	-0.0%	0.1%
AUS	2.8%	1.1%	4.0%	3.0%	-1.1%	1.9%
BRA	5.6%	0.1%	5.7%	1.5%	-0.1%	1.4%
CHL	8.8%	-4.1%	4.8%	3.4%	0.3%	3.7%
CHN	8.8%	-1.5%	7.4%	9.3%	-4.1%	5.2%
COL	6.0%	-2.2%	3.8%	3.3%	-1.6%	1.7%
IDN	7.4%	8%	15.4%	5.6%	1.2%	6.8%
IND	2.4%	1.4%	3.9%	5.1%	1.5%	6.6%
KOR	10.6%	1.5%	12.2%	10.1%	-2.7%	7.4%
MEX	7.3%	1.6%	8.9%	2.1%	1.0%	3.1%
SAU	4.3%	12.1%	16.4%	-0.5%	7.8%	7.3%
TUR	1.4%	0.9%	2.3%	4.8%	1.0%	5.8%
ZAF	2.2%	-0.4%	1.8%	1.3%	2.8%	4.0%
Median	*5.6%*	*0.9%*	*4.8%*	*3.3%*	*0.30%*	*4.0%*

Table S12: Decomposition factors Soviet Union crisis. Yearly average CO_2 emission and decomposition factors (GDP effect and Structural Change effect), for peak (left) and non-peak (right) countries before and after the Soviet

Union crisis (1990-91). Green (orange) indicates that the factor contribution change post-crisis was positive (negative) for decarbonisation. Sources:[55, 60, 61]

	Pre-crisis			Post-crisis		
	GDP	SC	CO2	GDP	SC	CO2
EST	3.8%	-3.2%	0.6%	0.2%	-5.0%	-4.8%
LTU	4.6%	-6.2%	-1.6%	-0.4%	-6.6%	-7.0%
LVA	2.5%	-8.4%	-5.9%	-1.0%	-5.5%	-6.5%
POL	1.3%	-0.1%	1.2%	3.2%	-6.5%	-3.3%
RUS	1.2%	0.2%	1.4%	2.9%	-4.8%	-1.9%
SVK	2.4%	-4.5%	-2.1%	1.7%	-4.5%	-2.8%
Median	*2.4%*	*-3.9%*	*-0.5%*	*0.9%*	*-5.1%*	*-3.9%*

	Pre-crisis			Post-crisis		
	GDP	SC	CO2	GDP	SC	CO2
ARG	-0.6%	1.2%	0.6%	3.3%	-1.5%	1.8%
AUS	3.4%	-1.2%	2.1%	3.2%	-0.8%	2.4%
BRA	2.3%	-0.5%	1.7%	2.5%	1.7%	4.2%
CHL	2.9%	1.0%	3.8%	5.9%	-1.0%	4.9%
CHN	9.9%	-4.3%	5.6%	10.2%	-5.8%	4.4%
COL	3.3%	-0.3%	3.0%	2.6%	-0.5%	2.1%
IDN	5.3%	0.4%	5.7%	3.8%	3.3%	7.2%
IND	5.6%	1.1%	6.7%	5.5%	-1.0%	4.5%
KOR	10.0%	-3.3%	6.7%	6.9%	-0.8%	6.1%
MEX	1.5%	1.3%	2.8%	3.1%	-0.5%	2.6%
NZL	2.1%	-1.2%	0.9%	3.1%	-0.4%	2.7%
SAU	-3.6%	10.2%	6.6%	2.2%	1.0%	3.2%
TUR	4.8%	1.4%	6.2%	2.8%	0.1%	2.9%
ZAF	1.7%	3.0%	4.7%	1.9%	-0.5%	1.4%
Median	*3.1%*	*0.7%*	*4.2%*	*3.1%*	*-0.5%*	*3.0%*

Table S13: Decomposition factors Global Financial Crisis (GFC). Yearly average CO_2 emission and decomposition factors (GDP effect and Structural Change effect), for peak (left) and non-peak (right) countries before and after the GFC (2008-09). Green (orange) indicates that the factor contribution change post-crisis was positive (negative) for decarbonisation. Sources:[55, 60].

	Pre-crisis			Post-crisis		
	GDP	SC	CO2	GDP	SC	CO2
AUT	2.5%	-1.6%	0.9%	1.1%	-1.9%	-0.8%
ESP	3.8%	-0.1%	3.7%	0.6%	-2.8%	-2.2%
FIN	3.6%	-2.4%	1.2%	0.3%	-3.0%	-2.7%
GRC	3.9%	-2.4%	1.6%	-2.4%	-1.7%	-4.1%
IRL	6.3%	-3.9%	2.4%	5.0%	-6.9%	-1.9%
ITA	1.5%	-0.7%	0.8%	-0.3%	-2.4%	-2.7%
JPN	1.2%	-0.3%	1.0%	0.6%	-1.9%	-1.3%
NLD	2.6%	-2.0%	0.6%	1.0%	-2.4%	-1.4%
NOR	2.4%	-2.3%	0.1%	1.2%	-1.6%	-0.5%
PRT	1.8%	-1.4%	0.3%	0.5%	-1.6%	-1.1%
SVN	4.4%	-3.5%	0.9%	1.0%	-3.1%	-2.1%
USA	2.9%	-2.2%	0.8%	1.8%	-3.0%	-1.2%
Median	*2.8%*	*-2.1%*	*0.9%*	*0.8%*	*-2.4%*	*-1.7%*

	Pre-crisis			Post-crisis		
	GDP	SC	CO2	GDP	SC	CO2
ARG	2.4%	0.0%	2.4%	0.6%	-0.1%	0.5%
AUS	3.5%	-1.4%	2.1%	2.5%	-2.8%	-0.2%
BRA	3.3%	-1.3%	1.9%	1.3%	-0.1%	1.2%
CAN	4.2%	-2.7%	1.4%	1.8%	-1.6%	0.2%
CHL	4.4%	-0.9%	3.5%	2.9%	-1.0%	1.9%
CHN	10.2%	-0.5%	9.7%	7.8%	-4.7%	3.1%
COL	3.3%	-4.0%	-0.6%	3.5%	-0.4%	3.1%
IDN	4.6%	1.3%	5.9%	5.3%	-0.8%	4.5%
IND	6.7%	-1.9%	4.9%	6.8%	-1.8%	5.0%
ISL	4.9%	-3.0%	1.9%	1.8%	-1.7%	0.1%
ISR	3.8%	-2.7%	1.1%	3.9%	-3.6%	0.3%
KOR	6.3%	-2.6%	3.7%	3.1%	-1.3%	1.8%
MEX	2.4%	0.3%	2.7%	1.9%	-1.5%	0.4%
NZL	3.8%	-1.7%	2.1%	2.6%	-2.3%	0.3%
SAU	2.9%	1.1%	4.0%	2.9%	0.3%	3.2%
TUR	4.4%	-0.1%	4.3%	4.8%	-1.6%	3.2%
ZAF	4.1%	-1.7%	2.4%	1.4%	-1.4%	-0.0%
Median	*4.1%*	*-1.4%*	*2.4%*	*2.9%*	*-1.5%*	*1.2%*

Supplementary References

1. Ciarli, T. and M. Savona, *Modelling the evolution of economic structure and climate change: a review.* Ecological economics, 2019. **158**: p. 51-64.
2. Savona, M. and T. Ciarli, *Structural changes and sustainability. A selected review of the empirical evidence.* Ecological economics, 2019. **159**: p. 244-260.
3. Mensch, G. and R. Schnopp, *Stalemate in Technology, 1925-1935: The Interplay of Stagnation and Innovation*. Vol. 11. 1980: Klett-Cotta.
4. Perez, C., *Unleashing a golden age after the financial collapse: Drawing lessons from history.* Environmental Innovation and Societal Transitions, 2013. **6**: p. 9-23.
5. Harris, J.M., *Green Keynesianism: Beyond standard growth paradigms*. 2013.
6. Cömert, M., *Revival of Keynesian Economics or Greening Capitalism:"Green Keynesianism".* Sosyoekonomi, 2019. **27**(42): p. 129-144.
7. Forster, P.M., et al., *Current and future global climate impacts resulting from COVID-19.* Nature Climate Change, 2020. **10**(10): p. 913-919.
8. Dupont, C., S. Oberthür, and I. von Homeyer, *The Covid-19 crisis: a critical juncture for EU climate policy development?* Journal of European Integration, 2020. **42**(8): p. 1095-1110.
9. Fioretos, O., T.G. Falleti, and A. Sheingate, *Historical institutionalism in political science.* The Oxford handbook of historical institutionalism, 2016: p. 4-28.
10. Del Río, P. and X. Labandeira, *Climate change at times of economic crisis.* Economía, 2009. **5**: p. 09.
11. Jalles, J.T., *Crises and emissions: New empirical evidence from a large sample.* Energy Policy, 2019. **129**: p. 880-895.
12. Loorbach, D.A. and R.L. Huffenreuter, *Exploring the economic crisis from a transition management perspective.* Environmental Innovation and Societal Transitions, 2013. **6**: p. 35-46.
13. Geels, F.W., *The impact of the financial–economic crisis on sustainability transitions: Financial investment, governance and public discourse.* Environmental Innovation and Societal Transitions, 2013. **6**: p. 67-95.
14. Geels, F.W., G.I. Pereira, and J. Pinkse, *Moving beyond opportunity narratives in COVID-19 green recoveries: A comparative analysis of public investment plans in France, Germany, and the United Kingdom.* Energy Research & Social Science, 2022. **84**: p. 102368.
15. Geels, F.W. and J. Schot, *Typology of sociotechnical transition pathways.* Research policy, 2007. **36**(3): p. 399-417.
16. Andreoni, V., *Estimating the European CO2 emissions change due to COVID-19 restrictions.* Science of the Total Environment, 2021. **769**: p. 145115.
17. Shammugam, S., et al., *Did Germany reach its 2020 climate targets thanks to the COVID-19 pandemic?* Climate Policy, 2022: p. 1-15.
18. Peters, G.P., et al., *Rapid growth in CO 2 emissions after the 2008–2009 global financial crisis.* Nature climate change, 2012. **2**(1): p. 2-4.
19. Le Quéré, C., et al., *Fossil CO 2 emissions in the post-COVID-19 era.* Nature Climate Change, 2021. **11**(3): p. 197-199.
20. Ray, R.L., et al., *What is the impact of COVID-19 pandemic on global carbon emissions?* Science of The Total Environment, 2022. **816**: p. 151503.
21. Siddiqi, T.A., *The Asian financial crisis—is it good for the global environment?* Global Environmental Change, 2000. **10**(1): p. 1-7.
22. Alsamara, M., et al., *Do economic downturns affect air pollution? Evidence from the global financial crisis.* Applied Economics, 2021. **53**(35): p. 4059-4079.
23. Ürge-Vorsatz, D., G. Miladinova, and L. Paizs, *Energy in transition: from the iron curtain to the European Union.* Energy Policy, 2006. **34**(15): p. 2279-2297.
24. Brizga, J., K. Feng, and K. Hubacek, *Drivers of CO2 emissions in the former Soviet Union: A country level IPAT analysis from 1990 to 2010.* Energy, 2013. **59**: p. 743-753.
25. Sobrino, N. and A. Monzon, *The impact of the economic crisis and policy actions on GHG emissions from road transport in Spain.* Energy Policy, 2014. **74**: p. 486-498.

26. Khan, I., et al., *A study of trilemma energy balance, clean energy transitions, and economic expansion in the midst of environmental sustainability: New insights from three trilemma leadership.* Energy, 2022. **248**: p. 123619.
27. Khan, I., D. Tan, and S.T. Hassan, *Role of alternative and nuclear energy in stimulating environmental sustainability: impact of government expenditures.* Environmental Science and Pollution Research, 2022. **29**(25): p. 37894-37905.
28. Arslan, H.M., et al., *Understanding the dynamics of natural resources rents, environmental sustainability, and sustainable economic growth: new insights from China.* Environmental Science and Pollution Research, 2022: p. 1-16.
29. Xu, X. and B.W. Ang, *Index decomposition analysis applied to CO2 emission studies.* Ecological Economics, 2013. **93**: p. 313-329.
30. Zhou, D., et al., *The role of structure change in driving CO2 emissions from China's waterway transport sector.* Resources, Conservation and Recycling, 2021. **171**: p. 105627.
31. Huang, F., et al., *Decomposition and attribution analysis of the transport sector's carbon dioxide intensity change in China.* Transportation Research Part A: Policy and Practice, 2019. **119**: p. 343-358.
32. Wang, M. and C. Feng, *Understanding China's industrial CO2 emissions: a comprehensive decomposition framework.* Journal of Cleaner Production, 2017. **166**: p. 1335-1346.
33. Yang, S., et al., *Regional variation in carbon emissions and its driving forces in China: An index decomposition analysis.* Energy & environment, 2013. **24**(7-8): p. 1249-1270.
34. Liu, N., Z. Ma, and J. Kang, *A regional analysis of carbon intensities of electricity generation in China.* Energy Economics, 2017. **67**: p. 268-277.
35. Yan, Q., et al., *Analysis of China's regional thermal electricity generation and CO2 emissions: decomposition based on the generalized Divisia index.* Science of the Total Environment, 2019. **682**: p. 737-755.
36. Wen, L. and Y. Hao, *Factor decomposition and clustering analysis of CO2 emissions from China's power industry based on Shapley value.* Energy Sources, Part A: Recovery, Utilization, and Environmental Effects, 2020: p. 1-17.
37. Wei, Y., et al., *Exploring the impact of transition in energy mix on the CO2 emissions from China's power generation sector based on IDA and SDA.* Environmental Science and Pollution Research, 2021. **28**(24): p. 30858-30872.
38. Zheng, B., et al., *Trends in China's anthropogenic emissions since 2010 as the consequence of clean air actions.* Atmospheric Chemistry and Physics, 2018. **18**(19): p. 14095-14111.
39. Chen, K., et al., *Study on the Influencing Factors of CO2 from the Perspective of CO2 Mitigation Potentials.* Sustainability, 2022. **14**(15): p. 9072.
40. Liu, B., et al., *Driving factors of carbon emissions in China: a joint decomposition approach based on meta-frontier.* Applied Energy, 2019. **256**: p. 113986.
41. De Oliveira-De Jesus, P.M., et al., *Multitemporal LMDI index decomposition analysis to explain the changes of ACI by the power sector in Latin America and the Caribbean between 1990–2017.* Energies, 2020. **13**(9): p. 2328.
42. De Oliveira-De Jesus, P.M., *Effect of generation capacity factors on carbon emission intensity of electricity of Latin America & the Caribbean, a temporal IDA-LMDI analysis.* Renewable and Sustainable Energy Reviews, 2019. **101**: p. 516-526.
43. Román, R., J.M. Cansino, and J.A. Rodas, *Analysis of the main drivers of CO2 emissions changes in Colombia (1990–2012) and its political implications.* Renewable Energy, 2018. **116**: p. 402-411.
44. Ščasný, M., B.W. Ang, and L. Rečka, *Decomposition analysis of air pollutants during the transition and post-transition periods in the Czech Republic.* Renewable and Sustainable Energy Reviews, 2021. **145**: p. 111137.
45. Cansino, J.M., A. Sánchez-Braza, and M.L. Rodríguez-Arévalo, *Driving forces of Spain′ s CO2 emissions: A LMDI decomposition approach.* Renewable and Sustainable Energy Reviews, 2015. **48**: p. 749-759.
46. Lamb, W.F., et al., *A review of trends and drivers of greenhouse gas emissions by sector from 1990 to 2018.* Environmental Research Letters, 2021.
47. Wang, H. and P. Zhou, *Assessing global CO2 emission inequality from consumption perspective: an index decomposition analysis.* Ecological Economics, 2018. **154**: p. 257-271.

48. Wang, H. and B. Ang, *Assessing the role of international trade in global CO2 emissions: An index decomposition analysis approach.* Applied Energy, 2018. **218**: p. 146-158.
49. Lamb, W.F., et al., *Countries with sustained greenhouse gas emissions reductions: an analysis of trends and progress by sector.* Climate Policy, 2022. **22**(1): p. 1-17.
50. Le Quéré, C., et al., *Drivers of declining CO 2 emissions in 18 developed economies.* Nature Climate Change, 2019. **9**(3): p. 213-217.
51. Sadorsky, P., *Energy related CO2 emissions before and after the financial crisis.* Sustainability, 2020. **12**(9): p. 3867.
52. Roinioti, A. and C. Koroneos, *The decomposition of CO2 emissions from energy use in Greece before and during the economic crisis and their decoupling from economic growth.* Renewable and Sustainable Energy Reviews, 2017. **76**: p. 448-459.
53. Kopidou, D. and D. Diakoulaki, *Decomposing industrial CO2 emissions of Southern European countries into production-and consumption-based driving factors.* Journal of Cleaner Production, 2017. **167**: p. 1325-1334.
54. Timma, L., T. Zoss, and D. Blumberga, *Life after the financial crisis. Energy intensity and energy use decomposition on sectorial level in Latvia.* Applied Energy, 2016. **162**: p. 1586-1592.
55. BP, *Statistical Review of World Energy, 71st Edition*. 2022.
56. OECD, *Indicators to Measure Decoupling of Environmental Pressure from Economic Growth*. 2002.
57. Naqvi, A. and K. Zwickl, *Fifty shades of green: Revisiting decoupling by economic sectors and air pollutants.* Ecological Economics, 2017. **133**: p. 111-126.
58. Tapio, P., *Towards a theory of decoupling: degrees of decoupling in the EU and the case of road traffic in Finland between 1970 and 2001.* Transport policy, 2005. **12**(2): p. 137-151.
59. Kallis, G., et al., *Research on degrowth.* Annual Review of Environment and Resources, 2018. **43**: p. 291-316.
60. World Bank, *World Development Indicators*, W. Bank, Editor. 2022.
61. Maddison Project Database, *Maddison style estimates of the evolution of the world economy. A new 2020 update*. 2020.
62. BP, *Statistical Review of World Energy 2021 | 70th edition.* 2021.
